# Dual Ionic Pathways in Semi‐Solid Electrolyte based on Binary Metal–Organic Frameworks Enable Stable Operation of Li‐Metal Batteries at Extremely High Temperatures

**DOI:** 10.1002/advs.202407018

**Published:** 2024-09-23

**Authors:** Minh Hai Nguyen, Nhat Minh Ngo, Byung‐Kook Kim, Sangbaek Park

**Affiliations:** ^1^ Department of Materials Science and Engineering Chungnam National University Daejeon 34134 Republic of Korea; ^2^ Energy Materials Research Center Korea Institute of Science and Technology (KIST) Seoul 02792 Republic of Korea

**Keywords:** lithium‐ion transport, Li‐metal batteries, metal–organic frameworks, semi‐solid‐state electrolyte, thermal stability

## Abstract

The rapid development of the electronics market necessitates energy storage devices characterized by high energy density and capacity, alongside the ability to maintain stable and safe operation under harsh conditions, particularly elevated temperatures. In this study, a semi‐solid‐state electrolyte (SSSE) for Li‐metal batteries (LMB) is synthesized by integrating metal–organic frameworks (MOFs) as host materials featuring a hierarchical pore structure. A trace amount of liquid electrolyte (LE) is entrapped within these pores through electrochemical activation. These findings demonstrate that this structure exhibits outstanding properties, including remarkably high thermal stability, an extended electrochemical window (5.25 V vs Li/Li^+^), and robust lithium‐ion conductivity (2.04 × 10^−4 ^S cm^−1^), owing to the synergistic effect of the hierarchical MOF pores facilitating the storage and transport of Li ions. The Li//LiFePO_4_ cell incorporating prepared SSSE shows excellent capacity retention, retaining 97% (162.8 mAh g^−1^) of their initial capacity after 100 cycles at 1 C rate at an extremely high temperature of 95 °C. It is believed that this study not only advances the understanding of ion transport in MOF‐based SSSE but also significantly contributes to the development of LMB capable of stable and safe operation even under extremely high temperatures.

## Graphical abstract

Integrating hierarchically porous metal‐organic frameworks with varying pore sizes establishes two distinct Li^+^ transport pathways for a trace amount of liquid electrolyte confined in these pores. It creates a semi‐solid‐state electrolyte characterized by high thermal and electrochemical stability, as well as Li‐ion conductivity, thereby guaranteeing stable cycling performance of Li//LiFePO_4_ cells even at extremely high temperatures like 95 °C.

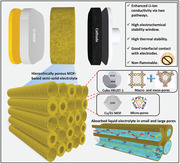

## 1 Introduction

Solid‐state Li‐metal batteries (SSLMBs) have attracted significant attention as potential solutions to enhance usage safety and elevate energy density in the next generation of rechargeable batteries.^[^ ^1^, ^2^, ^3^, ^4^, ^5^, ^6^, ^7^, ^8^ ^]^ The substitution of conventional liquid electrolytes (LEs) with solid‐state electrolytes (SSEs) offers superior thermal stability, a broader electrochemical stability window, and minimal electrolyte decomposition and volatilization.^[^ ^9^, ^10^, ^11^, ^12^, ^13^ ^]^ These inherent properties of SSEs significantly enhance battery performance and safety during operation.^[^ ^14^, ^15^, ^16^ ^]^ Moreover, the high density and outstanding mechanical properties of SSEs are advantageous in Li‐metal batteries, effectively mitigating the formation of Li dendrites.^[^ ^17^, ^18^, ^19^ ^]^ Nevertheless, SSLMBs still face challenges such as inadequate surface contact with electrodes, low ionic conductivity, high brittleness, and limited flexibility, which impede their widespread adoption and large‐scale production of macroscopic solid electrolytes.^[^ ^20^, ^21^, ^22^, ^23^, ^24^, ^25^, ^26^ ^]^

Recently, a semi‐solid‐state electrolyte (SSSE), created by integrating small quantities of liquid material, typically a liquid electrolyte (LE), into a solid electrolyte matrix, has exhibited significantly enhanced safety, durability, and electrochemical performance.^[^ ^17^, ^27^, ^28^, ^29^, ^30^, ^31^ ^]^ Such electrolyte configurations combine the merits of both solid and liquid electrolytes while circumventing their respective drawbacks.^[^ ^9^, ^32^ ^]^ In these systems, the host frameworks acts as excellent separator materials, effectively inhibiting lithium dendrite formation while maintaining high ionic conductivity and flexibility. Simultaneously, the inclusion of LE addresses issues such as poor electrode‐separator contact and slow interfacial kinetics, which are common contributors to battery performance degradation. Moreover, semi‐solid electrolytes provide a significantly safer operating environment compared to conventional liquid electrolytes, due to the minimal amount of LE incorporated within the host framework structure.^[^ ^33^, ^34^ ^]^ This innovative design shows great potential for the next generation of lithium batteries, offering the ability to meet stringent requirements for safety, stability, extended battery life, and robust performance even under harsh operating conditions, including high temperatures.

Among the host frameworks utilized in SSSEs, metal‐organic frameworks (MOFs) comprising metal ions and organic ligands have garnered significant attention in recent years owing to their well‐defined porous structures, ordered channels, and diverse compositions and morphologies.^[^ ^35^ ^]^ Notably, Huang et al. successfully fabricated MOF‐based ionic conductors with redistributed electronic density within channels, demonstrating their potential for high‐performance semi‐solid lithium metal batteries.^[^ ^36^ ^]^ Additionally, research by Liu's group highlighted the significant influence of MOF morphology, interstices, and cracks within MOF‐based separators on Li ionic conductivity and SSSE performance.^[^ ^37^ ^]^ Furthermore, Chang et al. developed a stable semi‐solid electrolyte based on copper hydroxide nanostrand MOF with a trace amount of LE, enabling the safe operation at 90 °C.^[^ ^38^ ^]^ These pioneering studies have revealed that the confinement of liquid electrolytes within the pore structures of MOFs occurs through two primary mechanisms: physical adsorption and chemical coupling between the anion moieties in the liquid electrolyte and the open metal sites of the MOFs.^[^ ^38^ ^]^ Additionally, the main mechanism of Li‐ion transport has been attributed to a hopping effect, driven by the capture and release of Li^+^ ions within the MOF channels.^[^ ^36^, ^37^ ^]^ Beyond these aspects, we also believe that the type and morphology of MOFs directly influence the adsorption and storage capacity of liquid electrolytes due to variations in pore size distribution. These variations significantly impact ion conductivity, ion transport mechanisms, and the thermal and electrochemical stability of MOF‐based semi‐solid‐state electrolytes. However, this relationship remains unclear and requires further investigation. Therefore, we further delved into the adsorption mechanism and physical properties of liquid electrolytes within MOFs of different pore sizes (micro‐, meso‐, and macro‐pores), presenting a novel mechanism for Li‐ion transport through hierarchical channels, thereby underscoring its advantages and applicability in Li‐ion batteries.

**Figure 1:**
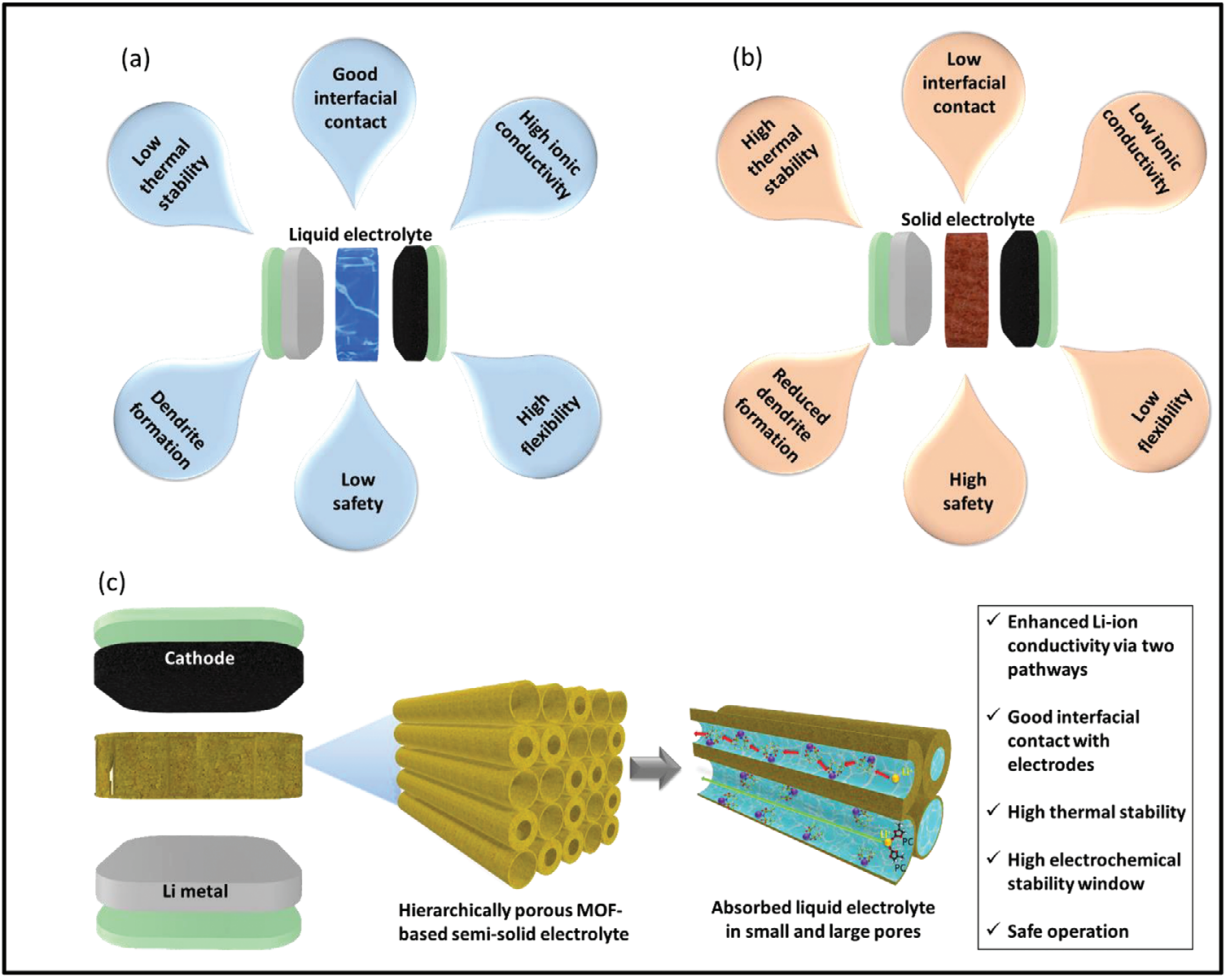
The advantages and disadvantages of a) conventional liquid electrolytes and b) solid‐state electrolytes. c) The outstanding properties of hierarchically porous MOF‐based semi‐solid electrolyte for a stable and high‐performance Li‐metal batteries.

In this study, we successfully fabricated SSSEs by integrating MOFs with hierarchical pore structures and sizes, wherein a small quantity of LE was introduced into the channels via electrochemical activation (**Figure** **1**). We systematically investigated the influence of hierarchical pore sizes and MOF morphology on the mechanism of Li‐ion transport through SSSE. Our findings revealed that the combination of HKUST‐1 cuboctahedral structures with rhombic dodecahedral Cu/Zn MOF resulted in outstanding Li^+^ transport efficiency through two distinct pathways within hierarchical pores. Furthermore, employing these MOF structures in a semi‐solid lithium battery with a LiFePO_4_ cathode (LFP) yielded remarkable specific capacity retention, with 98% and 97% of the initial value maintained after 100 cycles at room temperature and elevated temperature (60 °C), respectively. Particularly noteworthy is the safe and stable operation of our semi‐solid‐state LIB, even under high working temperatures of up to 95 °C (162.81 mAh g^−1^ after 100 cycles at 1 C, and 97% capacity retention). We believe that our investigation makes a significant contribution to future research on high‐performance MOF‐based SSSEs, facilitating safe and stable operation in high‐temperature environments.

## 2 Results and Discussion

**Figure 2:**
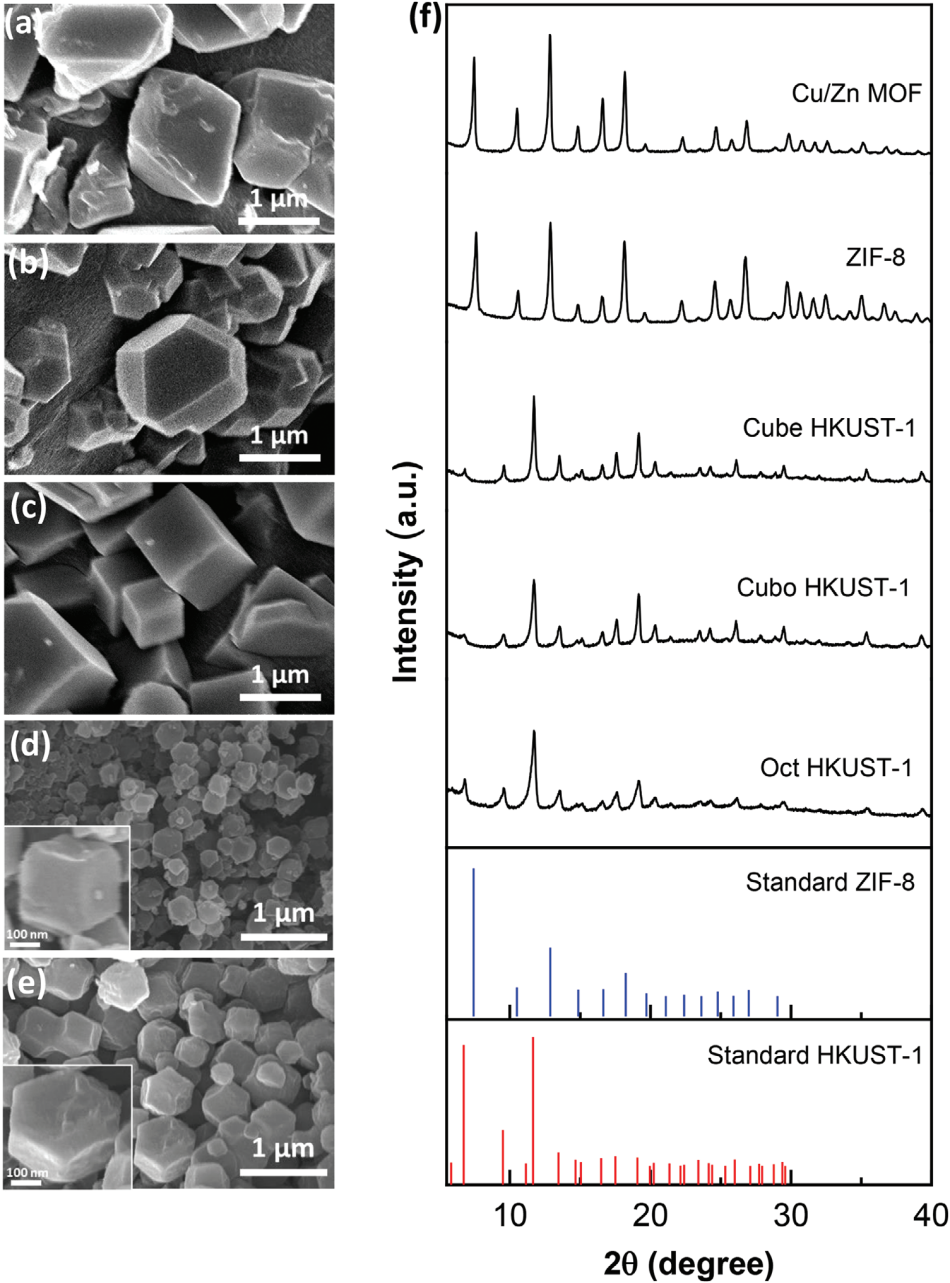
a–e) SEM images of as‐synthesized HKUST‐1 powders with morphologies of a) octahedron (Oct HKUST‐1), b) truncated cuboctahedron (Cubo HKUST‐1), and c) cube (Cube HKUST‐1), d) Zn‐based MOF (ZIF‐8), and e) Cu‐doped ZIF‐8 (Cu/Zn MOF). f) XRD patterns of various MOF powders.

Different crystal morphologies of HKUST‐1 were successfully fabricated through the addition of lauric acid with different concentrations during the solvothermal process (**Figure** **2**a–c). With increasing lauric acid concentration from 18.90 to 57.04 mmol, the crystal morphologies change from octahedron shape (denoted Oct HKUST‐1), truncated cuboctahedron (denoted Cubo HKUST‐1) and finally cube structure (symbolized as Cube HKUST‐1). The particle size of these samples was ≈1–2 µm. In the solvothermal reactions to fabricate HKUST‐1 MOF, lauric acid was reported to have the ability to act as a growth inhibitor to control the crystal growth facets in the {111} or {100} direction, causing morphological changes.^[^ ^39^ ^]^ ZIF‐8 and Cu‐doped ZIF‐8 (Cu/Zn MOF) were also successfully synthesized with rhombic dodecahedron morphology and particle size ≈300–500 nm (Figure 2d,e). The XRD patterns of ZIF‐8, Cu/Zn MOF, and three HKUST‐1 powders are presented in Figure 2f, showing good agreement with the standard MOFs patterns.

**Figure 3:**
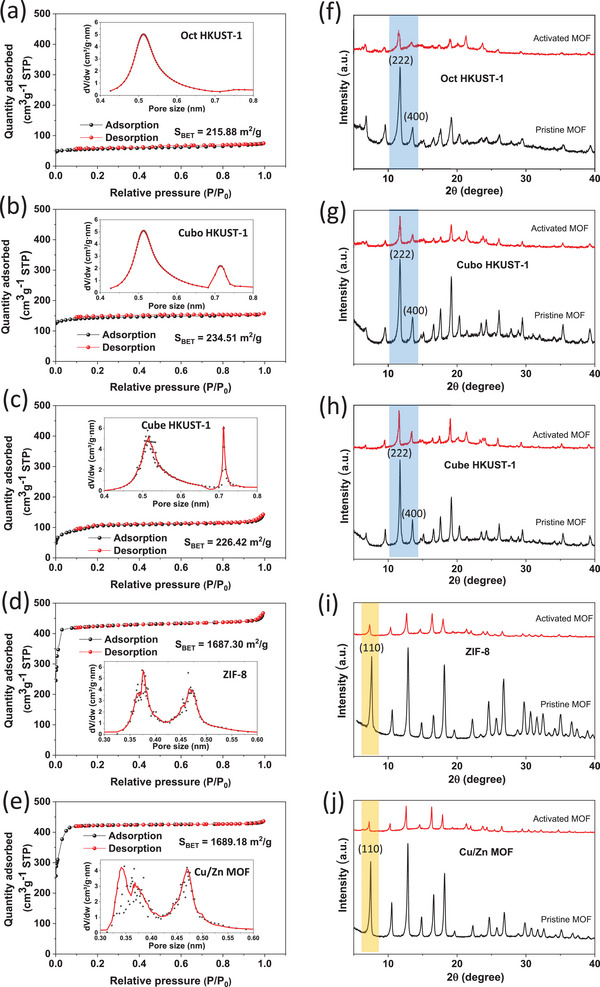
a–e) N_2_ adsorption‐desorption isotherms and pore size distributions and f–j) XRD patterns of a,f) Oct HKUST‐1, b,g) Cubo HKUST‐1, c,h) Cube HKUST‐1, d,i) ZIF‐8, and e,j) Cu/Zn MOF. XRD patterns of different MOFs were measured before and after electrochemical activation.

To investigate the infiltration of LiTFSI liquid electrolytes into the pore structures of different MOFs, we systematically analyzed pore size distribution, specific surface area, and lattice surface intensity variations within the MOF structures. Our analysis revealed distinct pore size distributions, with ZIF‐8 and Cu/Zn MOFs primarily exhibiting micro‐pores ranging from 0.34 to 0.46 nm, which are relatively smaller than those of the crystalline morphologies observed in HKUST‐1 (0.52–0.72 nm) (**Figure** **3**a–e). Consequently, there are variations in the BET‐specific surface area among the MOFs, with ZIF‐8 and Cu/Zn MOF powders boasting significantly higher specific surface areas (1690 m^2^ g^−1^) compared to HKUST‐1 samples (215–234 m^2^ g^−1^). This discrepancy can be attributed to the distribution of lattice faces within the MOF structures. Specifically, ZIF‐8 and Cu/Zn MOFs with rhombic dodecahedral morphology feature characteristic lattice planes, such as (110), with very small window sizes (≈0.34 nm),^[^ ^40^ ^]^ while HKUST‐1 samples exhibit characteristic lattice families {111} and {001} with larger window sizes of 0.46 and 0.9 nm,^[^ ^41^ ^]^ respectively. Observing the intensity change of MOF film peaks before and after electrochemical activation (Figure 3f–j), we noted a decrease in the intensity of XRD peaks, particularly for characteristic peaks and families of ZIF‐8 ((110)), Cu/Zn MOF ((110)), and HKUST‐1 ((222) and (400)) samples. These peaks exhibited a steeper decrease in intensity ratio compared to the remaining peaks, indicating the confinement and coordination of liquid electrolyte molecules within the pores of MOFs, especially those located on their characteristic lattice faces, resulting in a reduction in the intensity of acquired XRD signals from these facets. To further verify the effectiveness of LE confinement, the mass change of MOF‐based separators after electrochemical activation was carefully measured, and the mass percentage of LE confined within the MOF pores was also calculated. As shown in Table S1 (Supporting Information), the Cu/Zn MOF sample exhibited an LE retention capacity of up to 13.46% by mass, whereas the Cubo HKUST‐1 sample retained only 7.73%. The Cubo HKUST‐1@Cu/Zn MOF sample, which combines both MOFs, demonstrated an LE entrapment capacity of up to 11.41% (Table S1, Supporting Information). The larger specific surface area and pore density of the Cu/Zn MOF allowed this solid‐state electrolyte (SSSE) to adsorb and store a greater amount of LE in its pores compared to HKUST‐1. Furthermore, the data in Table S1 (Supporting Information) show a significant mass increase in all samples after activation, compared to simply soaking the SSSEs in pure LE. This indicates that electrochemical activation is highly efficient in introducing LE molecules into the MOF pores.

**Figure 4:**
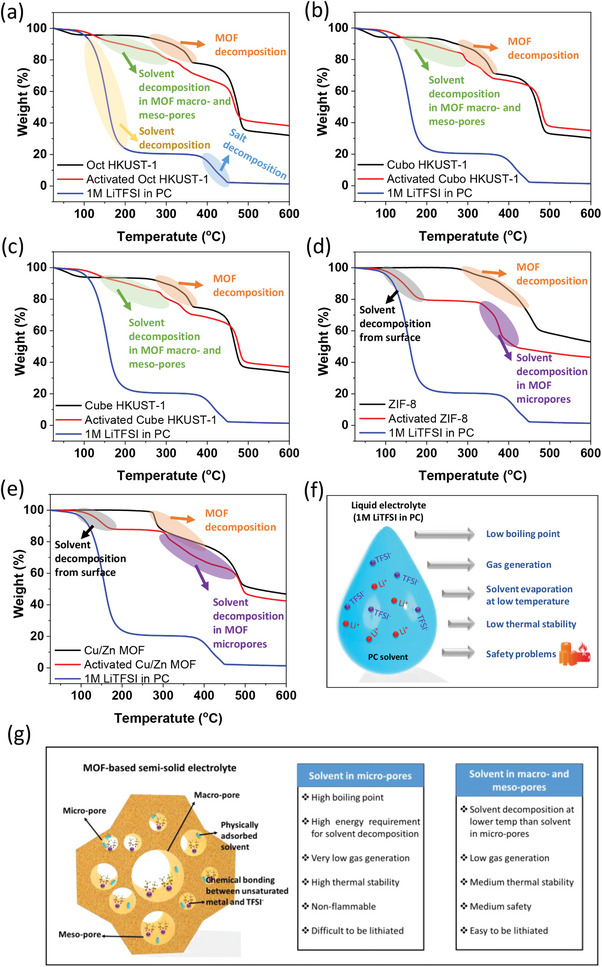
a–e) Thermogravimetric analysis (TGA) curves of the typical liquid electrolyte (1 m LiTFSI in PC) and different MOF films with and without electrochemical activation. f,g) Schematic illustration of f) the drawbacks of typical liquid electrolyte and g) the advantages and disadvantages of semi‐solid electrolytes based on MOFs with different pore sizes.

To analyze the thermal stability of MOF‐based semi‐solid electrolytes, thermogravimetric analysis (TGA) was performed over a temperature range from room temperature to 600 °C. Samples of HKUST‐1, ZIF‐8, and Cu/Zn MOF films without electrochemical activation exhibited thermal decomposition of MOF powders at temperatures exceeding 300 °C (marked in orange in **Figure** **4**a–e). The TGA curve of a typical liquid electrolyte (1 M LiTFSI in PC solvent) displayed two distinct weight losses: the first (highlighted in yellow) occurring ≈100–200 °C attributed to PC liquid solvent decomposition, while the second (highlighted in blue) was due to the decomposition of the LiTFSI salt (Figure 4a). In contrast, the TGA curves of semi‐solid electrolytes (MOF films after electrochemical activation) showed different trends (Figure 4a–e). For HKUST‐1 samples, gradual sample weight decomposition was observed between 150 and 300 °C (marked in green, Figure 4a–c), which was assigned to the thermal decomposition of the liquid solvent within the macro‐ and meso‐pores of these MOFs, significantly higher than conventional liquid electrolyte temperatures. In the case of ZIF‐8 and Cu/Zn MOF samples, an initial mass loss (shaded in gray) above 100 °C was likely due to residual liquid electrolyte on the film surface that was not completely removed before analysis (Figure 4d,e). Notably, a second TGA curve (highlighted in purple) appeared at a much higher temperature range, spanning over 300 °C to ≈500 °C (Figure 4d,e), corresponding to thermal decomposition of the liquid solvent within the micro‐pores of these MOFs. This highlights not only the presence but also the exceptional thermal stability of the liquid electrolyte within MOF pores, particularly within the micro‐pores of ZIF‐8 and Cu/Zn MOF samples. This significant enhancement in decomposition temperature can be attributed to the confinement of a very small amount of liquid electrolyte within nanostructured hosts provided by MOF pores.^[^ ^38^, ^42^, ^43^, ^44^ ^]^ As shown in Figure 4f, typical liquid electrolytes with large volumes exhibit relatively low boiling points, making them prone to evaporation and decomposition with increasing temperatures. Consequently, conventional liquid electrolytes often present safety risks, including gas production and potential explosions, particularly at elevated temperatures. In contrast, the thermal stability of liquid electrolytes confined within MOF micro‐pores is superior to that of electrolytes within larger pores (macro‐ and meso‐pores) and typical liquid electrolytes. The decomposition of minute amounts of liquid electrolyte within MOF micro‐pores requires greater heat input due to the nearly closed structure with a minimal open surface area (Figure 4g). Conversely, liquid electrolytes within MOF macro‐ and meso‐pores exhibit intermediate thermal stability, as their larger open windows facilitate LiTFSI salt and PC solvent migration and adsorption compared to MOF micro‐pores (Figure 4g).

**Figure 5:**
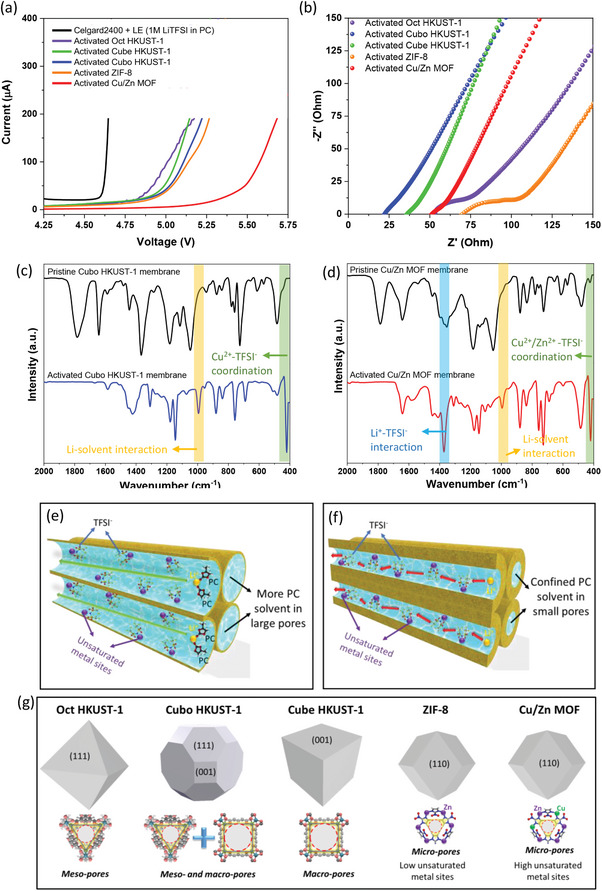
a) Linear sweep voltammetry (LSV) curves and b) Nyquist plots of different electrolytes. c,d) Fourier transform infrared spectrum (FT‐IR) of pristine and activated c) Cubo HKUST‐1 and d) Cu/Zn MOF membranes. e,f) Illustration of Li^+^ transport mechanisms e) in wide channels built by large pores, and f) in narrow channels built by small pores. g) Illustration representing the morphology and typical facets of MOF particles and the simulation of the theoretical window size on these facets.

The electrochemical properties of the prepared semi‐solid electrolyte were further investigated. Linear sweep voltammetry (LSV) revealed that the electrochemical stability window of semi‐solid electrolyte samples is significantly higher compared to that of the Celgard2400 separator in typical liquid electrolyte (LE) conditions (1 m LiTFSI in PC). The decomposition voltage reached 5.5 V for the activated Cu/Zn MOF sample and ranged from 4.8 to 5 V for the semi‐solid electrolyte samples, whereas the value for the Celgard 2400 combined with LE was only 4.6 V. This exceptional electrochemical stability of the synthesized semi‐solid electrolyte can be ascribed to the confinement effect of a small amount of liquid electrolyte within the nanoscale channels of the MOFs. This confinement raises the minimum energy required to decompose LEs trapped in MOF pores, ensuring the stable operation of MOF‐based semi‐solid electrolytes at higher voltages. Additionally, even unactivated MOF films exhibited superior electrochemical stability compared to the Celgard2400 separator when immersed in a bulk volume of LiTFSI liquid electrolyte (Figure S1, Supporting Information). However, since these films had not been electrochemically activated, the LE is not fully confined within the MOF pores, existing mainly in free form in the bulk solvent. As a result, the decomposition voltage remains lower than that of activated semi‐solid electrolytes. For the EIS measurement, HKUST‐1 samples generally exhibited superior ionic conductivity compared to ZIF‐8 and Cu/Zn MOF (**Figure** **5**b and Table S2, Supporting Information) owing to their structure featuring numerous macro‐ and meso‐pores capable of adsorbing a significant amount of LiTFSI salt and PC solvents. Consequently, the electrode surfaces have a higher probability of being thoroughly wetted in close contact with the semi‐solid electrolyte, resulting in a notable decrease in interfacial resistance and enhanced Li ion transport efficiency. Among the HKUST‐1 samples, the Cubo HKUST‐1 with a truncated cuboctahedron structure displayed the highest ionic conductivity (1.05 × 10^−4^ S cm^−1^) due to its hierarchical pore structure, with meso‐ and macro‐pores located on {111} and {001} facets, respectively, compared to the typical pore structures of Oct HKUST‐1 (meso‐pores on {111} facets) and Cube HKUST‐1 (macro‐pores on {001} facets) (Figure 5g). When comparing the two samples of ZIF‐8 and Cu/Zn MOF after activation, Cu/Zn MOF exhibited better ionic conductivity (4.18 × 10^−5^ S cm^−1^) (Figure 5b and Table S1, Supporting Information), which is believed to be due to its higher unsaturated metal sites resulting from Cu doping in the ZIF‐8 structure, leading to enhanced chemical adsorption of LiTFSI LE in MOF pores (Figure 5g). Figure S2 and Table S3 (Supporting Information) illustrated the ionic conductivity of cells containing bulk LE (1 m LiTFSI in PC) with MOF films without electrochemical activation, showing high ionic conductivity (>10^−4^ S cm^−1^) and minimal interfacial resistance. This can primarily be attributed to the influence of typical LE with high ionic conductivity and effective interfacial contact, highlighting that the impact of MOF films is minimal and not apparent without electrochemical activation.

Attenuated total reflectance Fourier transform infrared spectroscopy (ATR‐FTIR) was employed to explore the chemical interactions of LiTFSI LE inside MOF pores and the Li‐ion transport kinetics in MOF channels. Comparing the IR spectra of the Cubo HKUST‐1 film before and after electrochemical activation (Figure 5c), two new peaks were observed at 422 and 996 cm^−1^ in the IR spectrum of the activated Cubo HKUST‐1 sample. The first new peak at 422 cm^−1^ can be attributed to the overlap of Cu‐O lattice vibrations with the stretching vibration of S─N─S bond,^[^ ^45^ ^]^ indicating the interaction between TFSI anions and unsaturated Cu sites. Additionally, the second new peak at 996 cm^−1^ can be assigned to the Li^+^‐PC solvent interaction.^[^ ^38^ ^]^ In the IR spectrum of activated Cu/Zn MOF, new peaks similar to those in the Cubo HKUST‐1 sample are observed at 421 and 995 cm⁻¹, representing Cu^2^⁺/Zn^2^⁺‐TFSI⁻ coordination and Li⁺‐PC solvent interaction, respectively. Additionally, a prominent peak at 1372 cm⁻¹ is present, which is attributed to the interaction between Li⁺ and TFSI⁻. This leads to a reasonable assumption about the Li‐ion transport mechanism in MOF channels with different pore sizes as follows: MOFs with a broad pore size distribution (macro‐ and meso‐pores), such as Cubo HKUST‐1, allow easy ingress of both LiTFSI salt and PC solvent. TFSI^−^ radicals tend to interact and form ionic bonds with unsaturated Cu^2+^ sites, while Li^+^ is surrounded by a large amount of PC solvent molecules that are physically adsorbed into these large pores. This hinders the possibility of interaction between Li^+^ and TFSI^−^ in the channels, as confirmed by the FTIR analysis results (Figure 5c). Therefore, under the effect of the electric field force during the charge‐discharge process, Li⁺ moves in conjunction with the PC solvent through the large channels of Cubo HKUST‐1, similar to typical liquid electrolyte (Figure 5e). On the other hand, due to the narrow channel structure created by the micro‐pores of the Cu/Zn MOF, only a small amount of solvent is allowed to enter the channels, resulting in significantly lower Li^+^‐PC interaction than with large pores. Therefore, Li^+^ can be transported based on hopping via formation/breaking coordination with TFSI^−^ at unsaturated metal sites, as illustrated in Figure 5f.^[^ ^46^, ^47^ ^]^ This is further supported by the appearance of the Li^+^‐TFSI^−^ interaction peak in the IR spectrum of the activated Cu/Zn MOF membrane (Figure 5d). In conclusion, the study reveals that two main Li‐ion transport pathways in the channels of MOFs are influenced by pore size distribution. The transport mechanism of Li ions through two main pathways, as well as the interactions between MOFs and the liquid electrolyte (LE) inside MOFs, were further analyzed and elucidated using Raman spectroscopy, solid‐state ^7^Li magic‐angle‐spinning nuclear magnetic resonance (MAS NMR), ion transport activation energy, and lithium‐ion transference number. For more details, see Figures S3–S7 (Supporting Information). Each ion channel has both advantages and disadvantages. For channels that include macro‐ and meso‐pores, Li ions have better mobility with a direct transport path with the solvent under an electric field, leading to high ionic conductivity, as shown in the EIS measurement results (Figure 5b and Table S2, Supporting Information). However, because a large amount of PC solvent is contained in large pores, it leads to low thermal stability, the formation of a less stable SEI layer, and Li dendrite formation after long cycling. In the case of narrow channels with small‐sized pores (micro‐pores), the volume of PC solvent trapped by these pores is much lower than that of macro‐ and meso‐pores, thereby improving thermal stability and significantly reducing the possibility of Li dendrite formation. However, the limitation of these channels is that the ability to transfer ions is less flexible due to the hopping effect, and the ability to wet the surface of the electrodes is low because the PC solvent is tightly confined in small pores, leading to an increase in surface resistance and a decrease in ionic conductivity.

**Figure 6:**
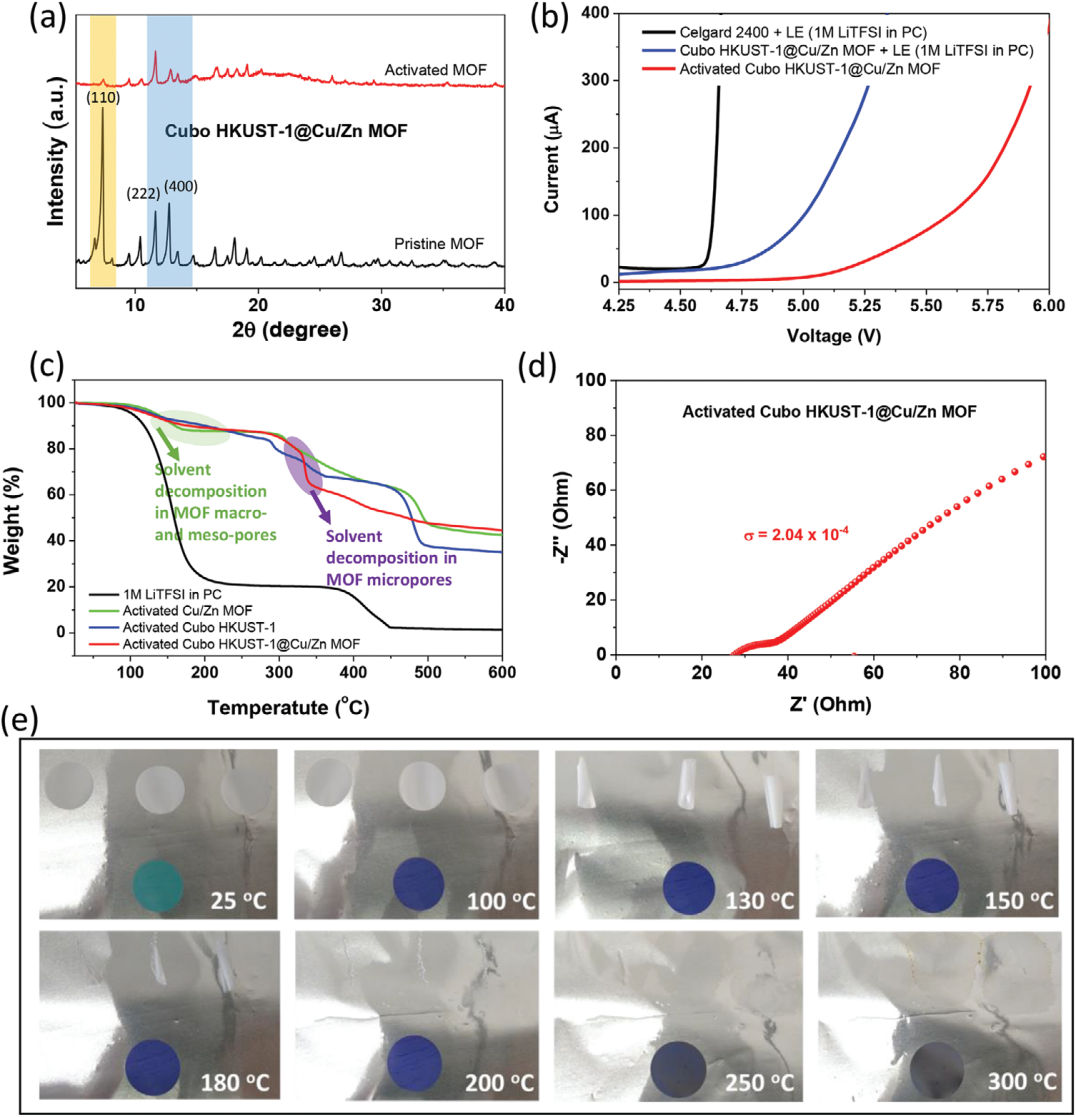
a) XRD patterns of Cubo HKUST‐1@Cu/Zn MOF before and after electrochemical activation. b) LSV curves typical liquid electrolyte and Cubo HKUST‐1@Cu/Zn MOF with and without activation. c) TGA curve of activated Cubo HKUST‐1@Cu/Zn MOF in comparison with typical liquid electrolyte, activated Cubo HKUST‐1, and activated Cu/Zn MOF. d) Nyquist plot of Cubo HKUST‐1@Cu/Zn MOF semi‐solid electrolyte. e) Thermal shrinkage images of the Celgard 2400 separator (white color) and Cubo HKUST‐1@Cu/Zn MOF semi‐solid electrolyte (blue color).

To harness the benefits and address the limitations of both channel types mentioned above, we fabricated a MOF‐based semi‐solid electrolyte with a hierarchical pore structure by combining Cubo HKUST‐1 and Cu/Zn MOF. These MOFs were mixed in a 1:1 mass ratio, and the semi‐solid electrolyte fabrication process mirrored that of other single MOFs. The resulting product was named Activated Cubo HKUST‐1@Cu/Zn MOF, showcasing high density and uniformity with a film thickness of ≈60 µm, as evidenced in the cross‐sectional SEM image (Figure S8, Supporting Information). XRD results depicted typical diffraction peaks of both Cubo HKUST‐1 and Cu/Zn MOF, such as the (110) peak of Cu/Zn MOF and the (222) and (400) peaks of Cubo HKUST‐1 (**Figure** **6**a). Significantly, these peaks all exhibited decreased intensity after the electrochemical activation process, indicating the confinement and coordination of liquid electrolyte molecules within predominantly hierarchical pores (micro‐, meso‐, and macro‐pores) on their characteristic lattice faces, resulting in the degradation of peak intensity. This phenomenon was further confirmed through TGA analysis (Figure 6c), where two mass losses observed at 150–250 °C and ≈350 °C were attributed to the thermal decomposition of the solvent in macro‐ and meso‐pores and micro‐pores of the MOF‐based semi‐solid electrolyte with a hierarchical pore structure. Owing to the confinement effect of liquid electrolyte molecules in a narrow structure, as mentioned previously, the electrochemical stability of the Activated Cubo HKUST‐1@Cu/Zn MOF sample was maintained at a high voltage of 5.25 V compared to the Celgard2400 combined with LE (4.6 V) and the Cubo HKUST‐1@Cu/Zn MOF + LE without activation (4.75 V) (Figure 6b). For the EIS measurement, the obtained ionic conductivity of Cubo HKUST‐1@Cu/Zn MOF SSSE also reached 2.04 × 10^−4^ S cm^−1^ (Figure 6d) due to the synergistic effect of hierarchical channels with the ability to transport Li ions along two different routes (Figure 5e,f). Thermal shrinkage of this semi‐solid electrolyte was evaluated and compared with Celgard2400 through heat treatment on the hot plate surface from 25 °C with a heating rate of 5 °C min^−1^ to 100, 130, 150, 180, 200, 250, and 300 °C and held for 30 min (Figure 6e). This is also an important parameter because it is directly related to the safety characteristics of the battery. The change in shape and size of membranes before and after heat treatment will indicate their thermal stability at different temperatures.^[^ ^48^ ^]^ It can be easily observed that Cubo HKUST‐1@Cu/Zn MOF SSSE has superior thermal shrinkage compared to Celgard2400 (Figure 6e). Even with heat treatment at 300 °C for 30 min, the area change of Cubo HKUST‐1@Cu/Zn MOF semi‐solid electroplate seems to be negligible (Figure 6e), while the size of Celgard2400 membrane begins to wrinkle at 130 °C and even melts completely at 200 °C (Figure 6e). Thermal discoloration above 250 °C may be due to the onset of decomposition of the MOF structure. This excellent temperature stability can be attributed to its semi‐solid structure with the main components being HKUST‐1 MOF and Cu/Zn MOF with thermal decomposition up to over 300 °C (TGA results, Figure 4a–e). Besides, the PVDF binder was also reported to have a decomposition temperature above 400 °C.^[^ ^49^ ^]^ The combustion tests also demonstrated that the binary MOF‐based SSSE exhibits superior fire resistance compared to the LE‐Celgard combination (Figure S9 and Videos S1 and S2, Supporting Information). This enhanced fire resistance is attributed to the stability of confined LE within the MOF pores. All of this proves that the Cubo HKUST‐1@Cu/Zn MOF sample has excellent thermal stability. This special heat resistance of our semi‐solid electrolyte can effectively prevent short circuits inside the cells, especially when operating at high temperatures.

**Figure 7:**
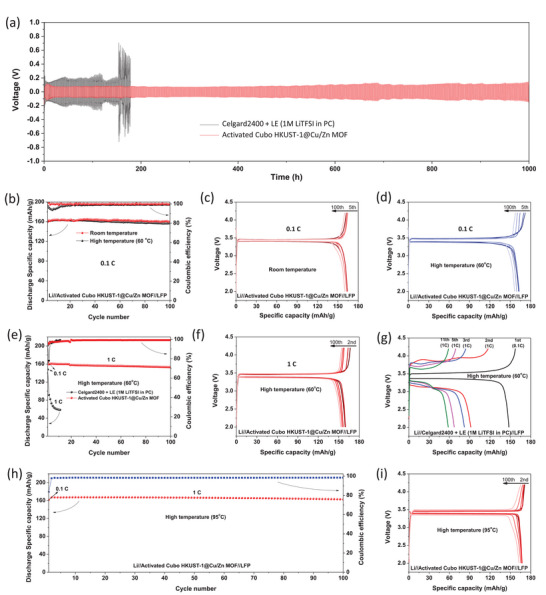
a) Galvanostatic cycling of Li//Li symmetric cells assembled with Celgard2400 combined with typical liquid electrolyte (LE) and Cubo HKUST‐1@Cu/Zn MOF semi‐solid electrolyte at current densities of 0.5 mA cm^−2^ at room temperature (RT). b–i) The cyclic performance of: b–d) Li/ Cubo HKUST‐1@Cu/Zn MOF/LFP at RT and high temperature (60 °C) with 0.1 C rate (0.09 mA cm^−2^); e–g) Li//LFP cells assembled with typical LE and Cubo HKUST‐1@Cu/Zn MOF semi‐solid electrolyte with 1 C rate (0.9 mA cm^−2^) at 60 °C; and h,i) Li/ Cubo HKUST‐1@Cu/Zn MOF/LFP at extremely high temperature (95 °C) with 1 C rate.

The MOF‐based semi‐solid electrolyte with a hierarchical pore structure has demonstrated an expanded electrochemical stability window and significantly enhanced ionic conductivity. Importantly, the prepared semi‐solid electrolyte exhibits a markedly improved decomposition temperature, ensuring safe operation even at high working temperatures. To validate this claim, we assembled Li//LFP half cells and Li//Li symmetric cells with the prepared solid electrolyte and operated them at room temperature (25 °C) and elevated temperatures (60 and 95 °C). Cells utilizing a conventional liquid electrolyte (1 m LiTFSI in PC) were also fabricated and measured for comparison. The Li plating/stripping behaviors of symmetric cells assembled with Cubo HKUST‐1@Cu/Zn MOF and Celgard 2400 combined with LE were analyzed by galvanostatic cycling at room temperature (**Figure** **7**a). Remarkably, the symmetric cell prepared with the hierarchically porous MOF membrane sustained long‐term cycling over 1000 h without short circuits at 0.5 mA cm^−2^ (areal capacity of 0.5 mAh cm^−2^), while the battery prepared with Celgard2400 and LE exhibited high and unstable overpotentials and developed short circuits after only 180 h. In addition, the Li metal surface detached from the symmetric cell using the Cubo HKUST‐1@Cu/Zn MOF separator maintained a smooth surface after 100 cycles, while the Li metal with the conventional Celgard 2400 separator clearly showed disordered and high‐density Li dendrites (see Figure S10, Supporting Information). This outstanding performance is greatly attributed to the advantages of the prepared MOF‐based semi‐solid electrolyte over conventional LE. In other comparisons, the symmetric cell assembled with activated Cu/Zn MOF and Cubo HKUST‐1 also displayed higher voltage polarization and poorer electrochemical stability (short circuit occurred after 390 and 220 h of cycling, respectively) compared to the cell with Cubo HKUST‐1@Cu/Zn MOF (Figures S11 and S12, Supporting Information). This further underscores the advantage of a hierarchical pore structure with two Li‐ion transport pathways compared to only one main pathway in a nonhierarchical pore structure. The Li//LFP coin cells utilizing prepared semi‐solid electrolytes were cycled at room temperature at a 0.1 C rate (0.09 mA cm^−2^) as well as various C rates from 0.1 C to 1 C, demonstrating excellent performance (Figure 7b,c; Figures S13 and S14a–d, Supporting Information). When tested at the same C rate at temperatures higher than 60 °C, the semi‐solid electrolyte‐based Li//LFP coin cell still delivered a high first discharge specific capacity (161.7 mAh g^−1^) and maintained good cycling stability at 156.6 mAh g^−1^ after 100 cycles, corresponding to a capacity retention of ≈97%, as shown in Figure 7b,d. Even at a 1 C rate (0.9 mA cm^−2^) and 60 °C, this cell operated stably with the capacity retention reaching ≈96% of the initial discharge specific capacity value (159.4 mAh g^−1^) after 100 cycles. In contrast, Li//LFP coin cells assembled with a typical liquid electrolyte showed very poor electrochemical performance under the same conditions (60 °C and 1 C rate) (Figure 7e,g). They suffered rapid capacity degradation after just a few cycles and remained at only 58.0 mAh g^−1^ after 11 cycles. To further challenge the prepared batteries' ability to operate under even harsher conditions, Li//LFP with Cubo HKUST‐1@Cu/Zn MOF semi‐solid electrolyte was further prepared and cycled at extremely high temperatures (95 °C) with a 1 C rate (Figure 7h,i). Surprisingly, the electrochemical performance of this cell remained very stable with the remaining capacity value reaching 97% of the initial discharge‐specific capacity (162.8 mAh g^−1^ after 100 cycles) (Figure 7h,i). Based on our literature review, this result represents the best battery performance obtained at such a high operating temperature.^[^ ^37^, ^38^, ^50^, ^51^ ^]^ Moreover, the stability analysis of the binary MOF SSSE, detached from this full cell after 100 cycles, showed no significant changes in morphology, structure, or chemical bonding compared to the pristine separator (see Figures S15–S17, Supporting Information). Conversely, the Li//LFP battery with Celgard 2400 and typical LE cannot operate at this very high temperature (95 °C), and cell failure occurs in the first cycle (Figure S18, Supporting Information). This is because the high cycling temperature (95 °C) approaches the thermal decomposition temperature of the bulk solvent in LE (100 °C) (TGA results, Figure 4a), while a small amount of LE is confined by the chemical coordination and physical adsorption in the pores of MOFs showed excellent thermal stability (TGA results, Figure 6c). In another comparison, the Li//LFP full cell with Cubo HKUST‐1 failed after only 25 cycles due to a micro short‐circuit, and the battery with Cu/Zn MOF reached 95% of capacity retention after 67 cycles and rapidly reduced due to LE depletion (see Figure S14e–h, Supporting Information). Li//LFP full cell with the Cubo HKUST‐1@Cu/Zn MOF separator was operated at 0.1C at low temperatures (2.5 °C) to further analyze the performance of batteries using the prepared SSSE under another harsh condition (Figure S19, Supporting Information). The results show that the battery performs relatively high, with an initial discharge capacity of 144.9 mAh g⁻¹. (see Figure S19, Supporting Information). The excellent performance of batteries assembled with binary MOFs based SSSE demonstrates the promising potential of semi‐solid electrolytes based on MOF composite with hierarchical pore structure in promoting battery performance as well as long lifetime even under extreme conditions.

## 3 Conclusion

This study successfully fabricated a MOF‐based semi‐solid electrolyte with a hierarchical pore structure by integrating two types of MOFs with distinct pore size distributions: truncated cuboctahedral HKUST‐1 and rhombic dodecahedral Cu/Zn MOF. The Li‐ion transport behavior in the prepared semi‐solid electrolytes containing different pore structures was thoroughly investigated. The results revealed two primary Li ion transport pathways in porous MOFs during cycling: direct transport through large channels (with macro‐ and meso‐pores) under the influence of the electric field, which contains numerous LE solvents, and migration through small channels (with micro‐pores) via the hopping effect. The semi‐solid electrolyte designed with a hierarchical pore structure, encompassing micro‐, meso‐, and macro‐pores, exhibited high ionic conductivity (2.04 × 10^−4^ S cm^−1^), a wide electrochemical stability window (5.25 V vs Li/Li^+^), and exceptional thermal stability owing to the synergistic effect of these MOF pores in storing and transporting Li ions. The lithium symmetric cell assembled with Cubo HKUST‐1@Cu/Zn MOF demonstrated outstanding cycling stability (over 1000 h at 0.5 mA cm^−2^), while the Li//LFP cell based on Cubo HKUST‐1@Cu/Zn MOF exhibited excellent capacity retention of 96% (153.3 mAh g^−1^) and 97% (162.8 mAh g^−1^) after 100 cycles at 1 C rate at high operating temperatures (60 °C) and very high temperatures (95 °C), respectively. We anticipate that the prepared semi‐solid electrolyte based on combined MOFs with a hierarchical pore structure in this study will significantly contribute to advancing the development and practical application of next‐generation batteries, ensuring stable operation and safety even at very high operating temperatures.

## 4 Experimental Section

### Preparation of different MOF Powders—Synthesis of HKUST‐1

HKUST‐1 with different morphologies was prepared according to a modified solvothermal synthesis.^[^ ^39^ ^]^ Copper(II) nitrate trihydrate (Cu(NO_3_)_2_·3H_2_O, 99%, Sigma‐Aldrich) (0.415 g, 0.68 mmol) and various amount of lauric acid (99%, Sigma‐Aldrich) (9.55 g, 18.90 mmol; 19.23 g, 38 mmol; and 28.75 g, 57.04 mmol) were mixed and dissolved in 50 mL of 1‐Butanol (99.9%, Sigma‐Aldrich). The linker solution was also prepared by dissolving 1,3,5‐benzenetricarboxylic acid (95%, Sigma‐Aldrich) (0.21 g, 0.38 mmol) in 50 mL of 1‐Butanol. This solution was then added dropwise over 30 min to prepare copper salt solution with magnetic stirring. The mixture was stirred for 30 min before transferring into Teflon‐lined stainless‐steel autoclave and heated at 140 °C for 2 h. The particles were collected by centrifugation (fixed‐angle rotor, 10 000 rpm, 10 min), washed with ethanol (99.9%, Thermo Fisher Scientific) three times, and dried at 90 °C. Finally, these powders were degassed by heating at 180 °C in a vacuum oven overnight and then stored in a desiccator for further processes. These collected powders were named as Oct HKUST‐1, Cubo HKUST‐1, and Cube HKUST‐1 based on the added lauric acid amounts of 9.55, 19.23, and 28.75 g, respectively. This synthesis process is depicted in Figure S20a (Supporting Information).

### Preparation of different MOF Powders—Synthesis of ZIF‐8

2.14 g of zinc nitrate hexahydrate (Zn (NO_3_)_2_·6H_2_O, 98%, Sigma‐Aldrich) and 4.64 g of 2‐Methylimidazole (2‐MIM, 99%, Sigma‐Aldrich) were dissolved in 50 mL methanol, respectively. Afterward, the 2‐MIM methanolic solution was added dropwise into Zn (NO_3_)_2_·6H_2_O methanolic solution followed by stirring for 15 h at room temperature. Finally, the product was collected by centrifugation (10 000 rpm, 10 min) and washed with methanol (99.8%, DAEJUNG) three times, and then dried at 180 °C oven overnight before storing in a desiccator for next steps. The detailed preparation process of ZIF‐8 is shown in Figure S20b (Supporting Information).

### Preparation of different MOF Powders—Synthesis of Cu‐Doped ZIF‐8 (Cu/Zn MOF)

A solution of the 0.66 g Zn(NO_3_)_2_.6H_2_O and 0.54 g Cu(NO_3_)_2_.3H_2_O (total 1 mmol) in 50 mL methanol and 2‐MIM (2.92 g) in the same volume of methanol were prepared separately. Then, these solutions were mixed by adding dropwise the 2‐MIM solution to the Cu^2+^‐Zn^2+^ solution. The synthesis was carried out at room temperature with stirring for 2 h. The powder was separated from the solution by centrifugation (10 000 rpm, 10 min) and washed with methanol (3 times × 30 mL). The obtained material was dried at 180 °C in a vacuum oven overnight and then stored at room temperature inside a desiccator before use. Figure S20c (Supporting Information) illustrates the detailed synthesis process of Cu/Zn MOF.

### Preparation of MOF Films

MOF powder was first mixed in a certain volume of acetone (99.5%, SAMCHUN) and ultrasonicated to obtain a homogeneous slurry. Then, Polyvinylidene fluoride (PVDF, 99.5%, MTI) solution (7.5 wt% in N, N‐Dimethylformamide (DMF, 99.5%, Sigma‐Aldrich) was added in MOF acetonic solution so that the MOF/PVDF weight ratio was 4/1. The obtained MOF slurries were stirred at room temperature without cap to mix the sample thoroughly and evaporate the remaining acetone and unnecessary organic solvent, finally resulting in sticky MOF ink. The MOF ink was then uniformly casted on the Al foil by a doctor blade and then dried at 120 °C in the vacuum oven overnight. The flexible MOF film was detached from Al foil by immersing MOF‐coated Al foil for a few minutes into methanol. The MOF film was then further pressed with 200 MPa of pressure to improve the density. Afterward, this film was hole‐punched to collect small MOF‐based separators with 18 mm of diameter. Finally, these MOF separators were degassed again at 180 °C overnight in the vacuum oven before storing them into the desiccator for further usage. The detailed fabrication process of MOF films is shown in Figure S21 (Supporting Information).

### Preparation of Semi‐Solid Electrolytes

First, 1.44 g of Lithium Bis(Trifluoromethanesulfonyl) Imide (LiTFSI, 99.9%, Sigma‐Aldrich) was mixed homogeneously with 5 mL Propylene Carbonate (PC, 99.7%, Sigma‐Aldrich) solvent to prepare 1 m LiTFSI liquid electrolyte. To prepare the semi‐solid electrolyte, MOF films were electrochemically activated in the Li//MOF//Li symmetric cell with 1 m LiTFSI liquid electrolyte under 0.5 mA cm^−2^ for ten cycles (Figure S22, Supporting Information). Afterward, the MOF‐based semi‐solid electrolytes were finally obtained by disassembling MOF films from the symmetric cell and removing the excess liquid electrolyte on the surface of films. The prepared semi‐solid electrolytes were stored inside glove box for further use to fabricate various batteries in this study.

### Preparation of LFP Cathode

The LFP cathode was fabricated using a blade‐casting method. LiFePO_4_ powder (MTI), Super P (TIMCAL), and PVDF were mixed with a weight ratio of 8:1:1 in N‐methyl‐2‐pyrrolidone (NMP, 99.5%, SAMCHUN) using a ball‐miller. The obtained slurry was cast onto a carbon‐coated Al foil and heated at 110 °C under vacuum for 10 h. The mass loading of active materials was ≈5.3 mg cm^−2^. LFP cathode was then cut into electrode slices with a diameter of 18 mm for coin‐cell fabrications and kept in Ar‐filled glovebox to avoid humidity.

### Cell Assembly and Electrochemical Measurements

All CR2032 coin cells were assembled in Ar‐filled glove box to avoid the effect of moisture and oxygen. To prepare for Li//LFP half cells, the obtained semi‐solid electrolytes were tightly attached to LFP cathode and Li plates (MTI) by a physical pressing process before assembling in CR2032 coin cells (Figure S23a,b, Supporting Information). Other half cells using 40 µL of typical liquid electrolyte (1 m LiTFSI in PC) and the Celgard2400 (MTI) as a separator were also prepared for comparison (Figure S23c, Supporting Information). These cells were cycled between 2.0 and 4.2 V vs Li/Li^+^ at various current densities starting from 0.09 (0.1 C) to 0.9 mA cm^−2^ (1 C), using a computer‐controlled battery cycler (Neware) at room temperature and high temperature (60 and 95 °C). To assemble Li//Li symmetric cells, two Li foils were applied as electrodes, and MOF‐based semi‐solid electrolytes or Celgard2400 were used as separators (Figure S24a,b, Supporting Information). These asymmetric cells were cycled at 0.5 mAh cm^−2^ (0.5 mA cm^−2^ for 1 h), using the battery cycler at room temperature. The Linear sweep voltammetry (LSV) measurement was conducted under the control of a potentiostat (IVIUM potentiostat/galvanostat, IVIUM technologies, Eindhoven, Netherlands) in the voltage range of 3–6 V. The Li//separator&electrolyte//SS spacer coin cell was used for LSV measurement. The Electrochemical Impedance Spectroscopy (EIS) test was carried out using an impedance spectrometer (Solartron SI 1260, AMETEK SI) in the frequency range of 1 Hz–10 MHz at various temperatures from 25 to 70 °C. The symmetric coin cells with two stainless steel electrodes were applied for the ionic conductivity measurement. Lithium‐ion transference number was measured by the potentiostat with an applied DC voltage of 10 mV and EIS measurement in a lithium symmetric cell incorporating prepared MOF SSSE and commercial Celgard2400 in LE (1 m LiTFSI in PC). EIS was tested before and after the DC polarization conducted by amperometric technique. The $t_{Li^{+}}$ value was calculated according to Bruce's equation: $t_{Li^{+}}$= I_s_ (∆V–I_0_R_0_) / (I_0_ (∆V–I_s_R_s_), where ∆V was the polarization voltage (10 mV), I_0_ was the initial current, I_s_ was the steady state current, R_0_ was the initial resistance, and R_s_ was the steady state total resistance

### Characterizations

The morphologies of MOF powders and MOF films were observed using a field emission scanning electron microscope (FESEM, SU7000_HITACHI) at an accelerating voltage of 10 kV. The energy‐dispersive X‐ray spectroscope (Ultim Max65, Oxford) was used to investigate the elemental distribution. To analyze the crystal structure of MOF films before and after electrochemical activation, X‐ray diffractometer (D8 ADVANCE, Bruker) was used with a Cu‐Kα radiation source (λ = 1.54 Å) and a scanning speed of 5° min^−1^ in the 2 θ range of 5–40°. X‐ray photoelectron spectroscopy (XPS) of MOF‐based SSSE before and after 100 cycling operations were carried out on Thermo Scientific KAlpha+ spectrometer with Al‐Kα excitation. The specific surface area (SSA) and pore size distribution of different MOFs were determined by the BET Surface Area Analyzer (Micromeritics, ASAP 2020). Attenuated total reflection Fourier‐transform infrared (ATR‐FTIR) was carried out on a vacuum infrared spectrometer (VERTEX 80v, Bruker) coupled with Platinum Diamond ATR to detect traces of LiTFSI liquid electrolytes in MOF pores after electrochemical activation through specific coordinations. To investigate the thermal stability of MOF‐based semi‐solid electrolytes and typical LiTFSI liquid electrolyte, thermogravimetric analysis was conducted on a thermal analyzer (Mettler‐Toledo) with a temperature range of 25–600 °C and 10 °C/min of heating rate. Raman spectra of MOF‐based SSSEs and pure LiTFSI were collected by Raman analysis equipment (Model: HEDA; Manufacturer: WEVE). Samples were excited by an argon ion laser at a wavelength of 532 nm with a grating of 1200 gr. The scattered light was collected in a direction of 90° to the incident light. ^7^Li Solid‐state magic‐angle spinning (MAS) NMR measurements, along with T_1_ relaxation analysis, were performed to study the Li‐ion diffusion kinetics in activated MOF‐based separators. The ^7^Li MAS NMR spectra were recorded using a 3.2 mm solid‐state NMR probe at a spinning rate of 15 kHz on a 600 MHz NMR spectrometer (VARIAN, INOVA). T_1_ measurements were conducted using an inversion recovery pulse sequence with an array of 6–10 data points. T_1_ was then calculated using the following equation: I = I_0_ (1 – 2e^−t/T1^), where I was the peak intensity at time t, I_0_ was the saturation intensity, and T_1_ was the longitudinal (or spin‐lattice) relaxation time.

## Conflict of Interest

The authors declare no conflict of interest.

## Supporting information



Supporting Information

Supporting Information

Supporting Information

## Data Availability

The data that support the findings of this study are available from the corresponding author upon reasonable request
