# Supplementary material for: Dual Ionic Pathways in Semi‐Solid Electrolyte based on Binary Metal–Organic Frameworks Enable Stable Operation of Li‐Metal Batteries at Extremely High Temperatures
Source: Adv Sci (Weinh). 2024 Sep 23;11(43):2407018. doi: 10.1002/advs.202407018 (PMC11578376; doi:10.1002/advs.202407018)
Supplement: Supplementary file 1 — Supporting Information [file ADVS-11-2407018-s001.docx]

Supporting Information

**Dual ionic pathways in semi-solid electrolyte based on binary metal-organic frameworks enable stable operation of Li-metal batteries at extremely high temperatures**

*Minh Hai Nguyen, Nhat Minh Ngo, Byung-Kook Kim, and Sangbaek Park^*^*

**Table S1.** Comparison of the loading mass of liquid electrolyte (LE) in pores of different MOF-based SSSEs.

| **Sample** | **Mass of pristine samples (g cm^-2^)**  **(A)** | **Mass of samples after dipping in LE (g cm^-2^)**  **(B)** | **Mass of samples after electrochemical activation (g cm^-2^)**  **(C)** | **Mass of LE in pores (g cm^-2^)**  **(D = C - B)** | **Loading percent of LE in pores (%)**  **(E = 100*D/C)** |
| --- | --- | --- | --- | --- | --- |
| Cubo HKUST-1 | 0.01065 | 0.01318 | 0.01429 | 0.00110 | 7.73 |
| Cu/Zn MOF | 0.00877 | 0.01169 | 0.01351 | 0.00182 | 13.46 |
| Cubo HKUST-1@Cu/Zn MOF | 0.01021 | 0.01623 | 0.01832 | 0.00209 | 11.41 |


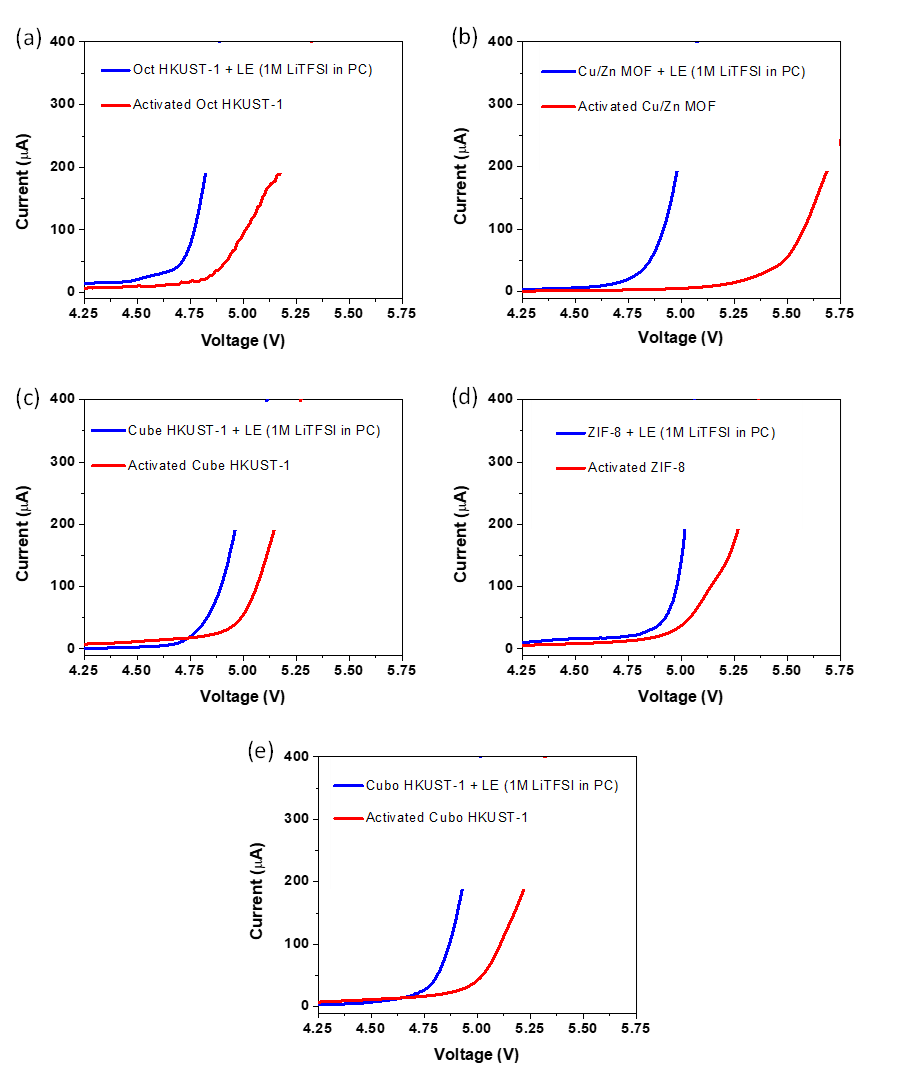


**Figure S1**. Linear sweep voltammetry (LSV) curves of activated and unactivated (pristine MOF combined with typical LE) (a) Oct HKUST-1, (b) Cubo HKUST-1, (c) Cube HKUST-1, (d) ZIF-8, and (e) Cu/Zn MOF.

**Table S2**. The ionic conductivity of different MOF-based semi-solid electrolytes

| **Sample** | **Ionic conductivity (S cm^-1^)** |
| --- | --- |
| Activated Oct HKUST-1 | 3.05 x 10^-5^ |
| Activated Cubo HKUST-1 | 1.05 x 10^-4^ |
| Activated Cube HKUST-1 | 6.06 x 10^-5^ |
| Activated ZIF-8 | 3.65 x 10^-5^ |
| Activated Cu/Zn MOF | 4.18 x 10^-5^ |


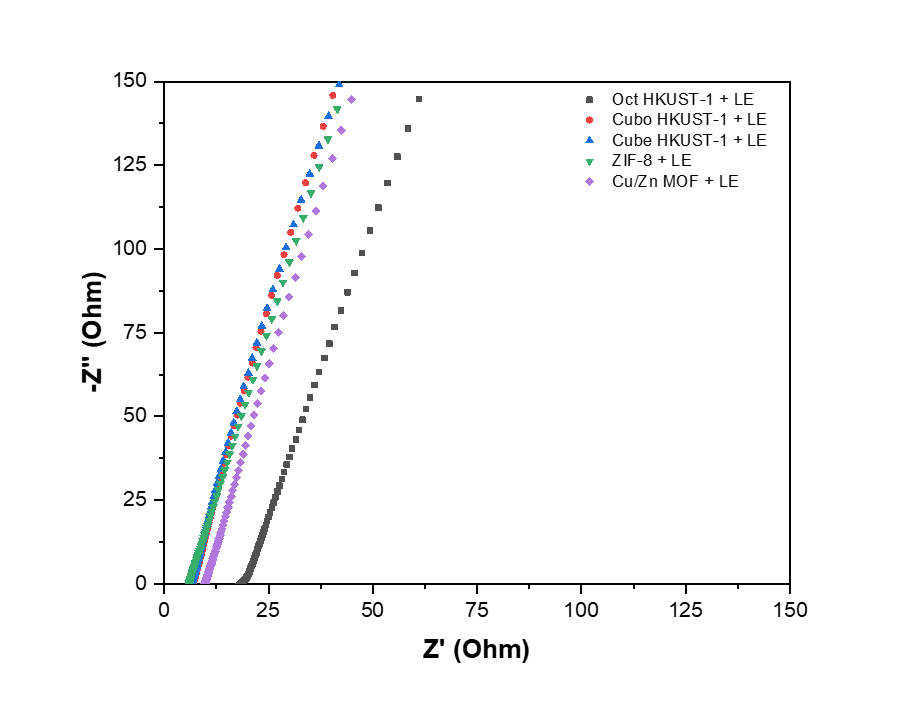


**Figure S2**. Nyquist plots of different MOF membranes in LE without electrochemical activation.

**Table S3**. The ionic conductivity of unactivated MOF films in typical LE.

| **Sample** | **Ionic conductivity (S cm^-1^)** |
| --- | --- |
| Oct HKUST-1 + LE | 1.23 x 10^-4^ |
| Cubo HKUST-1 + LE | 4.30 x 10^-4^ |
| Cube HKUST-1 + LE | 3.84 x 10^-4^ |
| ZIF-8 + LE | 6.58 x 10^-4^ |
| Cu/Zn MOF + LE | 2.55 x 10^-4^ |

To further substantiate our conclusion regarding the dual Li-ion transport pathways in the MOF-based SSSEs, we explored the chemical environment and coordination of the liquid electrolyte (LE) within the MOF pores, as well as the Li-ion transport kinetics in the nanochannels of the MOFs, using Raman spectroscopy and solid-state ^7^Li magic-angle-spinning nuclear magnetic resonance (MAS NMR) (Figures S3, 4). A characteristic strong Li-TFSI peak at 748 cm^-1^ was observed in the pure LiTFSI salt sample, which significantly decreased and shifted to 826 cm^-1^ in the Cubo HKUST-1 SSSE (Figure S3), indicating an interaction between Li^+^ and the PC solvent in the unactivated LE. The presence of a strong peak at 1008 cm^−1^ and a broad peak at 278 cm^−1^ suggests that Li^+^-PC-TFSI^-^ interactions dominate within the pores of Cubo HKUST-1, indicating that the primary Li-ion transport pathway is direct transport through the large channels of Cubo HKUST-1. In contrast, the Cu/Zn MOF with smaller channels exhibits significant enhancement of Li-TFSI-Metal^n+^ interactions, attributed to strong sub-nanoscale confinement effects. This is confirmed by the strong peaks of Li^+^-TFSI^-^-Cu^2+^ and Li^+^-TFSI^-^-Zn^2+^ at 278 cm^−1^ and 385 cm^−1^, respectively (Figure S3), indicating that ion transport primarily occurs via the hopping effect in the small channels of Cu/Zn MOF. The absence of a Li^+^-PC peak in Cu/Zn MOF also suggests that its electrochemical activation efficiency is significantly better than that of Cubo HKUST-1.

**Figure S3.** Raman spectra of pure LiTFSI, Cubo HKUST-1, and Cu/Zn MOF SSSEs.

We also carried out the solid NMR measurement to further clarify the Li-ion transportation properties of prepared SSSE (Figures S4, 5). MAS NMR ^7^Li spectra of Cubo HKUST-1 SSSE show two distinct chemical shifts to lower and higher frequencies (Figure S4), corresponding to the shielding effect induced by the Lewis acid-base interaction between Cubo HKUST-1 and Li ions (activated LE within MOF pores) and LiTFSI in the PC solvent (unactivated LiTFSI), respectively. For the Cu/Zn MOF SSSE, the microporous channels effectively retain activated LE, as evidenced by the appearance of a strong LiTFSI peak in the MOF pores at a lower frequency (Figure S4). We further studied the mobility of Li^+^ in MOFs using spin-lattice relaxation time (T1) analysis in solid ^7^Li NMR (Figures S5). T1 measurements, based on the Bloembergen–Purcell–Pound model,^[1]^ assess species mobility by simulating the spin-lattice expansion caused by the thermal motion of the nucleus through energy exchange with its surroundings. A shorter T1 time indicates faster species mobility. Our results show that activated Li@MOFs in this study exhibit high Li^+^ mobility, with T1 times ranging from 0.029 s to 0.224 s (Figures S 5b, c), which are shorter compared to pure LiTFSI salt (T1 = 13.55 s) (Figure S5a) and some MOF and covalent–organic framework (COF) based electrolytes reported in other studies, such as Li@MOF-5 (T1 = 1.008 s),^[2]^ Li@Zn-MOF-74 (T1 = 0.578 s),^[2]^ TpPa-SO_3_Li (T1 = 2.76 s),^[3]^ and LiClO_4_@COF-5 (T1 = 1.91 s).^[4]^ Compared to HKUST-1, the Cu/Zn MOF SSSE exhibits a significantly shorter T1 time of 0.036 s, which is attributed to the more uniform and faster diffusion of Li^+^ ions via the hopping effect in the high-density nanochannels of its microporous structure, as opposed to the more random and slower diffusion of Li^+^ ions in the low-density macrochannels of the macroporous structure of HKUST-1.

**Figure S4.** 7Li Solid-state MAS NMR spectra of pure LiTFSI, activated Cubo HKUST-1, and activated Cu/Zn MOF.

**Figure S5.** Inversion-recovery plots, fitted curves, and calculated T_1_ of (a) pure LiTFSI, (b) Cubo-HKUST-1 SSSE, and (c) Cu/Zn MOF SSSE.


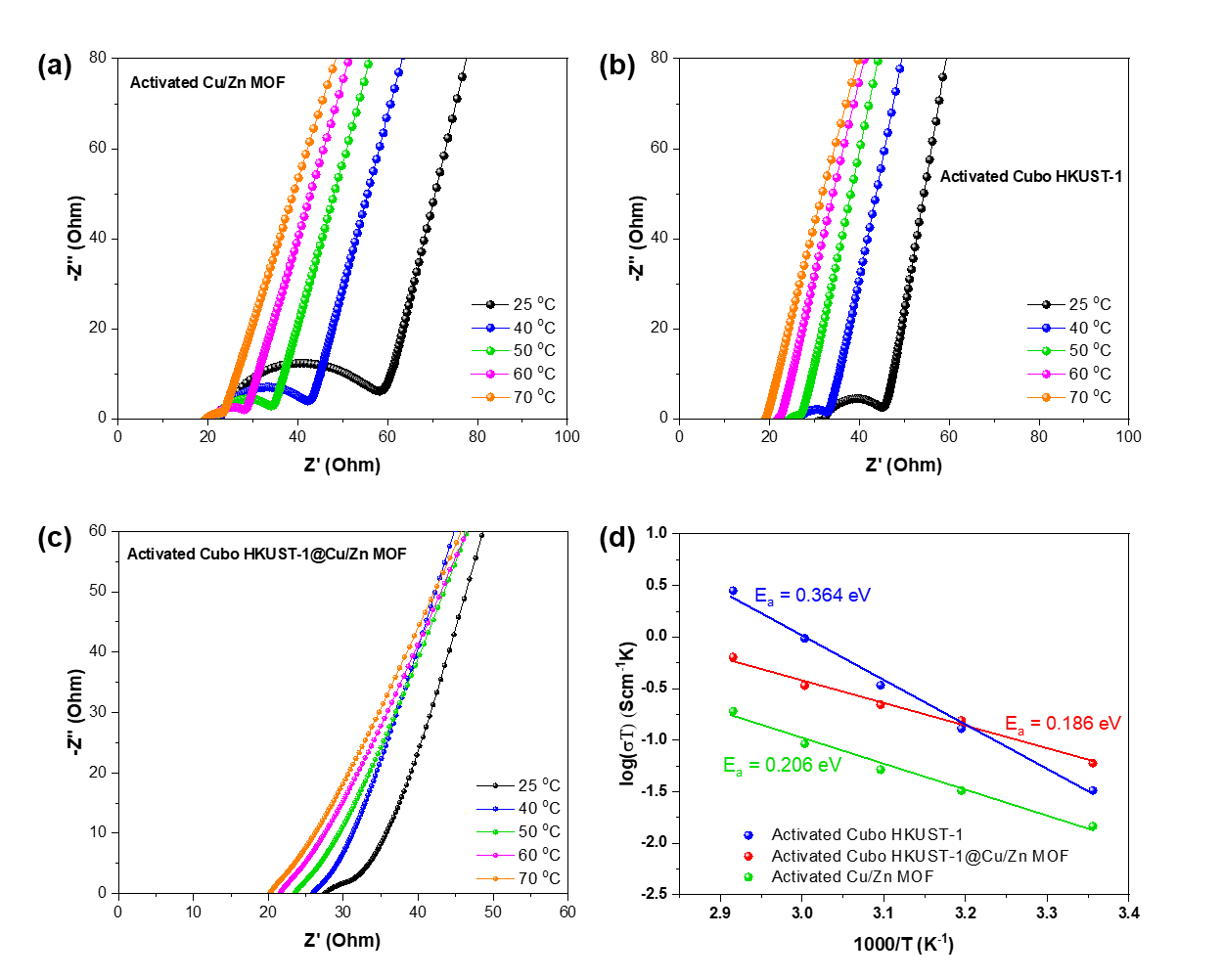
To further analyze ionic transport in different MOF-based semi-solid electrolytes (SSSEs), we conducted Electrochemical Impedance Spectroscopy (EIS) measurements across a temperature range from room temperature (25 °C) to 70 °C and calculated the ion transport activation energy using Arrhenius plots, as shown in Figures S6a-d. The linear shape of all plots indicates that no structural or compositional changes occurred in the MOF-based separators during the measurements, confirming the thermal stability of these SSSEs within the 25–70 °C range. Notably, the activation energy of the binary MOF-based SSSE (0.186 eV) is significantly lower than that of the HKUST-1 (0.364 eV) and Cu/Zn MOF (0.206 eV) SSSEs. This reduction in activation energy can be attributed to the homogeneous and rapid Li-ion transport mechanism facilitated by the two reciprocating channels in the nanoporous structure of the binary Cubo HKUST-1@Cu/Zn MOF, which enhances Li^+^ transfer kinetics.

**Figure S6.** Nyquist plots of activated (a) Cu/Zn MOF, (b) Cubo HKUST-1, and (c) Cubo HKUST-1@Cu/Zn MOF at various temperatures from 25 to 70 ^o^C, and (d) their corresponding Arrhenius plots.

We also measured the lithium-ion transference number ($t_{{Li}^{+}}$) of the binary MOF-based SSSE at room temperature using the Bruce–Vincent method^[5]^ and compared it with that of the Celgard2400 separator in a liquid electrolyte (1M LiTFSI in PC). The Li-ion transference number increased significantly from 0.387 with the liquid electrolyte (Figures S7a) and commercial Celgard2400 separator to 0.663 with the Cubo HKUST-1@Cu/Zn MOF SSSE (Figures S7b). This improvement can be explained by the immobilization of TFSI^-^ anions by the open metal sites of both MOFs and the size-selective ion sieving of these anions by the small pore apertures of the Cu/Zn MOF, resulting in a relatively high Li-ion transference number for the prepared SSSE.

**Figure S7.** Nyquist plots of the Li//Li symmetric cell of (a) Celgard2400 and (b) Cubo HKUST-1@Cu/Zn MOF SSSE before and after polarization (the insets are the current–time curves at 10 mV polarization).


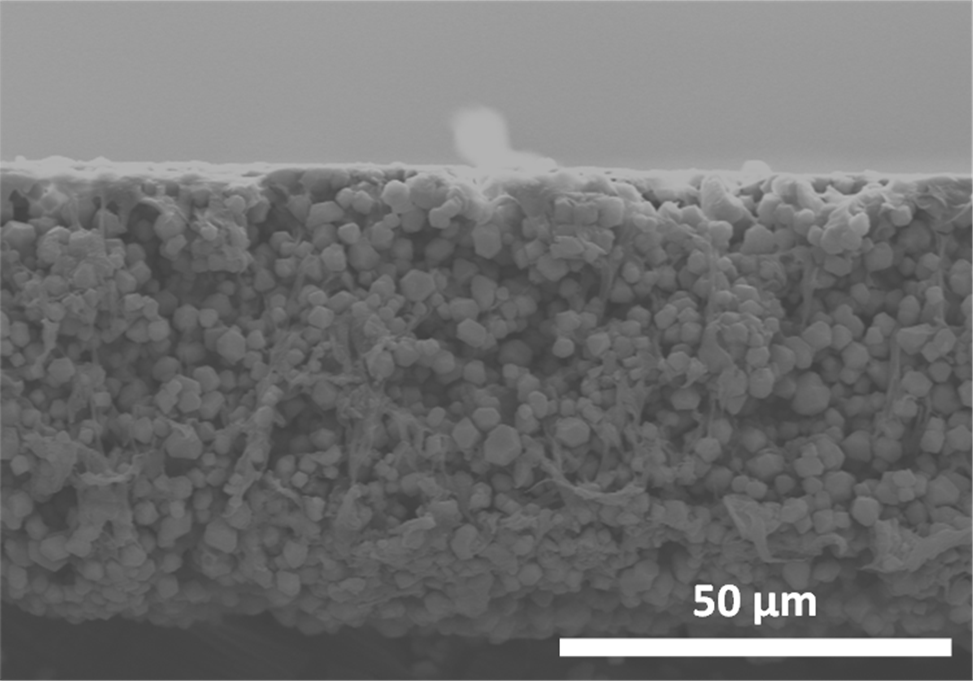


**Figure S8**. Cross-sectional SEM image of Cubo HKUST-1@Cu/Zn MOF semi-solid electrolyte.

**Figure S9.** Combustion tests of Celgard2400 in LE (1M LiTFSI in PC) and Cubo HKUST-1@Cu/Zn MOF semi-solid electrolyte

We analyzed the surface morphology of Li metal extracted from symmetric cells using our designed MOF-based semi-solid-state electrolyte (SSSE) after 100 cycles, employing Scanning Electron Microscopy (SEM). We compared these results with Li metal from cells assembled with Celgard2400 and liquid electrolyte (LE). The SEM images clearly show disordered and high-density Li dendrites on the Li metal surface with the conventional Celgard2400 separator (Figures S10b, d). In contrast, the Li metal surface with the Cubo HKUST-1@Cu/Zn MOF separator maintained a smooth surface (Figures S10a, c). This indicates the effectiveness of the SSSE in suppressing the formation and growth of Li dendrites, thereby reducing the risk of short circuits during battery operation. The uniform ion fluxes through the nanochannels in the ordered porous structure of the Cubo HKUST-1@Cu/Zn MOF significantly contributed to uniform Li deposition on the Li metal surface. Conversely, the commercial Celgard2400 separator in LE, which lacks uniform nanochannels, led to disordered, non-uniform ion fluxes and slower transport rates, promoting the formation of Li dendrites.

**Figure S10.** SEM images of Li metal surfaces detached from the Li//Li symmetric cell using (a, c) Cubo HKUST-1@Cu/Zn MOF and (b, d) commercial Celgard2400 separator.


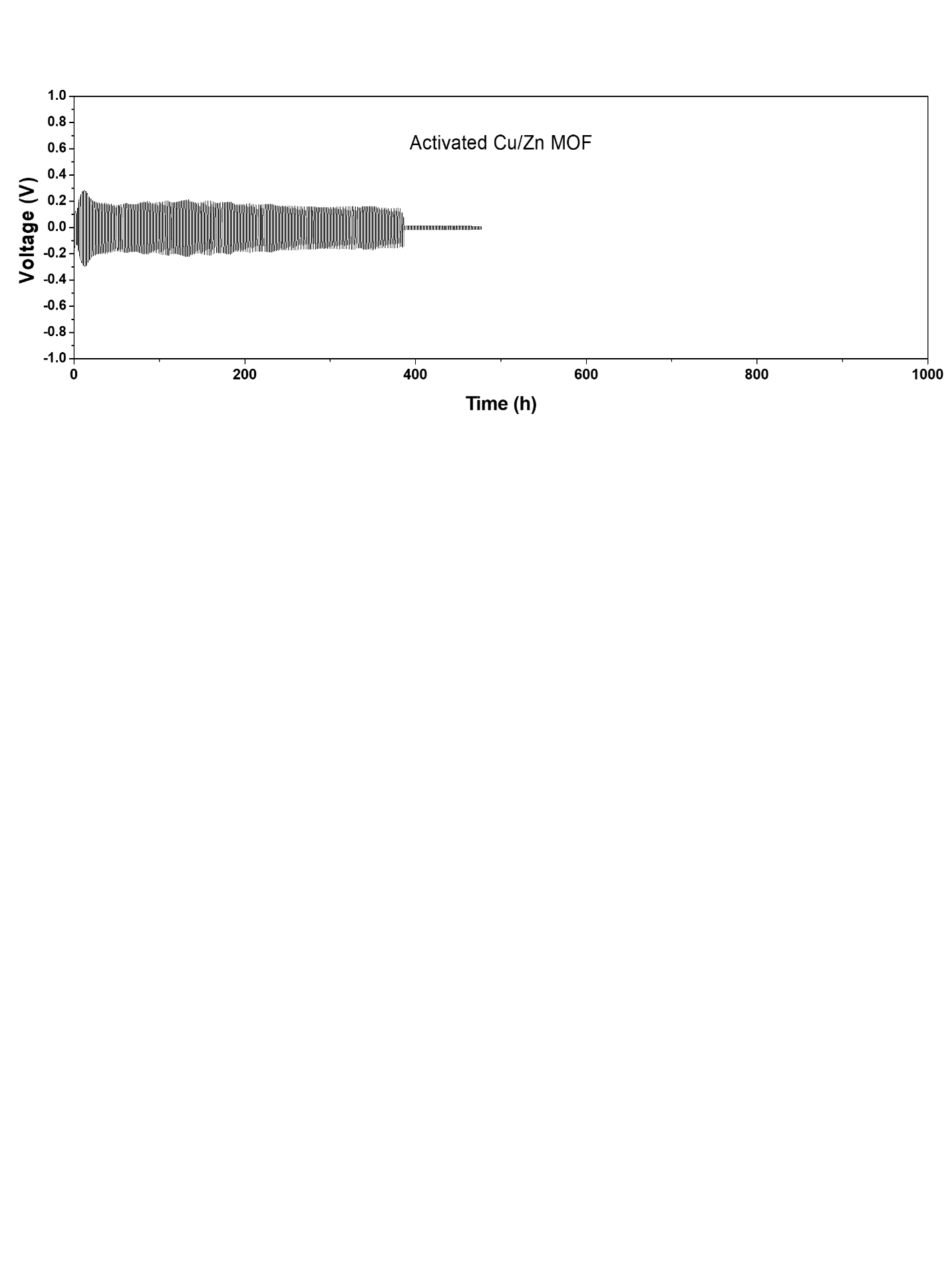


**Figure S11**. Galvanostatic cycling of Li//Li symmetric cells assembled with Cu/Zn MOF semi-solid electrolyte.

**Figure S12.** Galvanostatic cycling of Li//Li symmetric cells assembled with Cubo HKUST-1 semi-solid electrolyte.

**Figure S13**. The cyclic performance of: Li/ Cubo HKUST-1@Cu/Zn MOF/LFP at room temperature with various C rates (0.1, 0.3, 0.5 and 1 C).

We measured additional Li//LFP full cell with Cubo HKUST-1 at high temperature, and the result showed that the battery failed after only 25 cycles due to a micro short-circuit (Figures S14e, f). In contrast, the full cell with Cu/Zn MOF demonstrated better stability, with capacity retention reaching 95% after 67 cycles, followed by a rapid decline due to increased voltage polarization (Figures S14g, h). This decline is attributed to LE depletion in the SSSE under high-temperature conditions, rather than a short-circuit phenomenon as seen in the cell with Cubo HKUST-1. These findings highlight the significant influence of pore size distribution on battery performance. Cubo HKUST-1, with its larger pore size, allows Li ions to be transported mainly through the PC environment and the influence of the electric field, which is similar to ion transport in LE. In contrast, the Cu/Zn MOF, with its smaller pore distribution, facilitates Li-ion transport through the hopping effect, enabling more uniform ion distribution and reducing the likelihood of Li dendrite formation and short-circuiting. However, the physically absorbed LE in the micropores of the Cu/Zn MOF is gradually consumed, eventually becoming insufficient to wet the electrode, leading to increased resistance and rapid capacity loss. These phenomena occur more rapidly and are more easily observed at high temperatures (95°C). Nevertheless, full cells with these MOFs still operate stably after 100 cycles with high capacity (~150 mAh g⁻¹) at RT and continue running (Figures S14a-d).


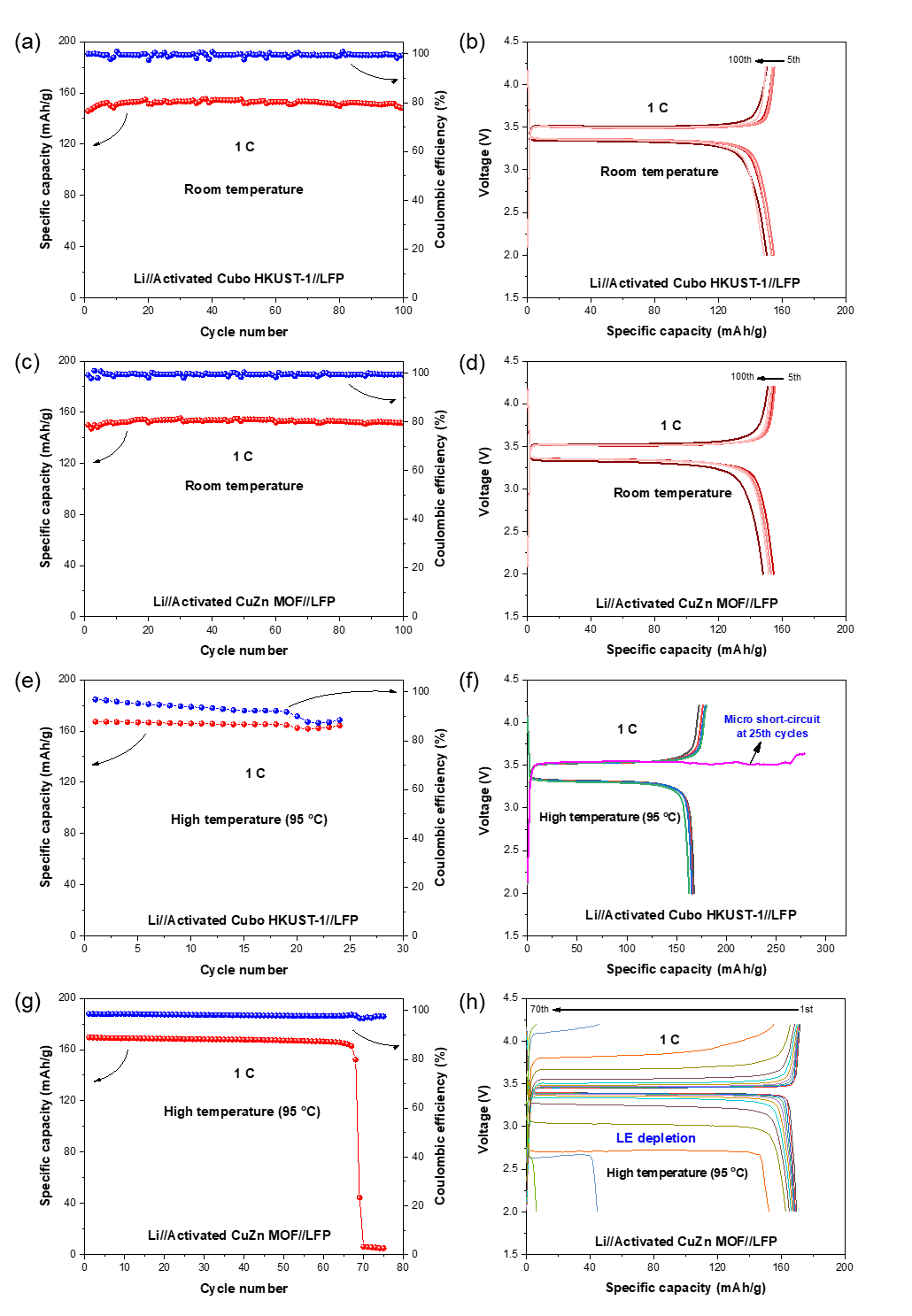
**Figure S14.** The cyclic performance of: Li/Cubo HKUST-1/LFP at (a, b) RT and (e, f) high temperature (95 ^o^C) with 1 C rate; and Li/Cu/Zn MOF/LFP at (c, d) RT and (g, h) high temperature (95 ^o^C) with 1 C rate.

To further assess the durability of the Cubo HKUST-1@Cu/Zn MOF semi-solid electrolyte, we assembled and tested Li//LFP full cells with this SSSE at 1C and high temperature (95 °C). After 100 cycles, we disassembled the cells and subjected the SSSEs to SEM, XRD, and XPS analyses, comparing them with pristine samples. Cross-sectional SEM images (Figure S15) reveal that after 100 cycles, the Cubo HKUST-1@Cu/Zn MOF SSSE shows no increase in volume or thickness, no cracks, no formation of Li dendrites, and no significant changes in the morphology of the MOFs. Additionally, XRD analysis indicates that the crystal structure of the SSSE remains largely unchanged after 100 cycles at high temperature, with the typical diffraction peaks of activated Cu/Zn MOF and Cubo HKUST-1 still clearly observed (Figure S16). XPS analysis of the Cubo HKUST-1@Cu/Zn MOF SSSE, focusing on the elements Li 1s, Cu 2p, Zn 2p, F 1s, and O 1s, also shows only minor changes after operation (Figures S17a-e). The slight change in the intensity of the C 1s peaks (Figure S17f) can be attributed to the gradual decomposition of LE after 100 cycles at high temperatures. These results demonstrate that the binary MOF-based SSSEs exhibit remarkable durability even under extremely harsh conditions.

**Figure S15.** Cross-sectional SEM images of (a) pristine Cubo HKUST-1@Cu/Zn MOF semi-solid electrolyte and (b) the separator after 100 cycles.


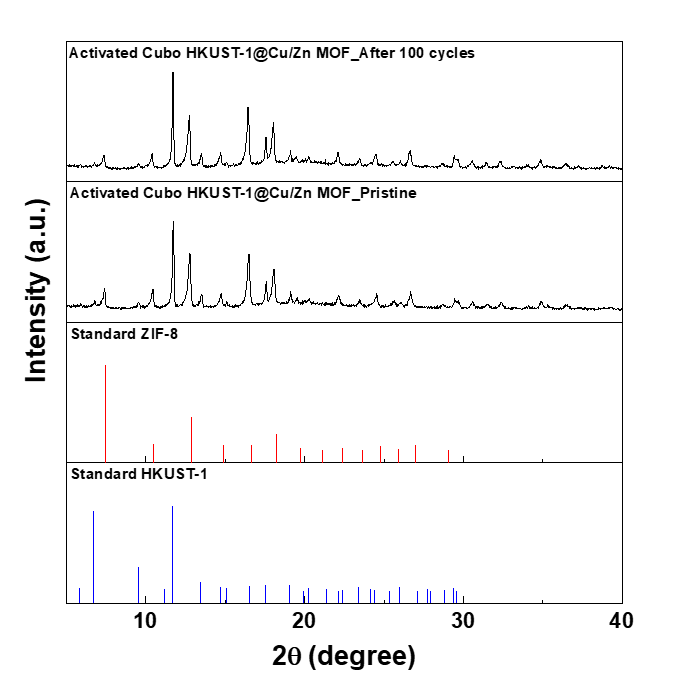


**Figure S16.** XRD spectra of pristine Cubo HKUST-1@Cu/Zn MOF semi-solid electrolyte and this separator after 100 cycles.

 **Figure S17.** XPS spectra of different elements of pristine Cubo HKUST-1@Cu/Zn MOF semi-solid electrolyte and those after 100 cycles.


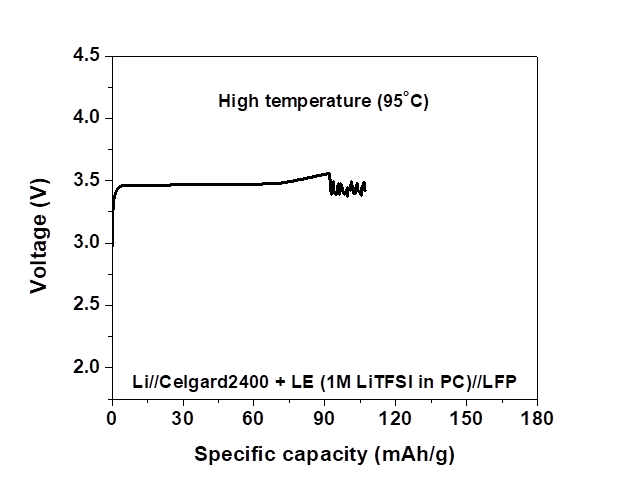


**Figure S18**. Galvanostatic charge–discharge (GCD) curve of Li//LFP cells assembled with typical LE at 95 ^o^C with 0.1 C rate.

To further evaluate the performance of batteries using the prepared SSSE under harsh conditions, we tested Li//LFP full cells with the Cubo HKUST-1@Cu/Zn MOF separator at low temperatures (2.5°C) and a charge/discharge rate of 0.1C (Figure S19). The results show that the battery performs relatively high, with an initial discharge capacity of 144.9 mAh g⁻¹. The capacity loss at low temperatures is expected and is primarily due to the slowing of internal reactions and the reduced Li⁺ diffusion coefficient in both the electrodes and the electrolyte. However, the capacity drop is not severe, which may be attributed to the properties of the propylene carbonate (PC) solvent and the unique Li⁺ transport mechanism in the MOF-based SSSE structure. The PC solvent has a high dielectric constant (64.92) compared to other common commercial solvents such as dimethoxyethane (DME, 7.2), dimethyl carbonate (DMC, 3.107), and diethyl carbonate (DEC, 2.805).^[6]^ This high dielectric constant facilitates excellent salt dissociation, enhances Li⁺ dissolution, and improves the transfer kinetics of Li⁺. Additionally, the very low freezing point of PC (-48.8°C)^[7]^ makes it suitable for low-temperature battery operation. The combination of PC's excellent properties and the high ion conductivity through the dual channels in the MOF pores further enhances cell performance even at low temperatures. Further analysis of cell performance, battery durability, and ion transport via MOF-based SSSEs at low and very low temperatures will be conducted in our future studies.

**Figure S19.** The galvanostatic charge–discharge (GCD) curves of Li//Cubo HKUST-1@Cu/Zn MOF //LFP full cell at low temperature (2.5 ^o^C) with 0.1 C rate.


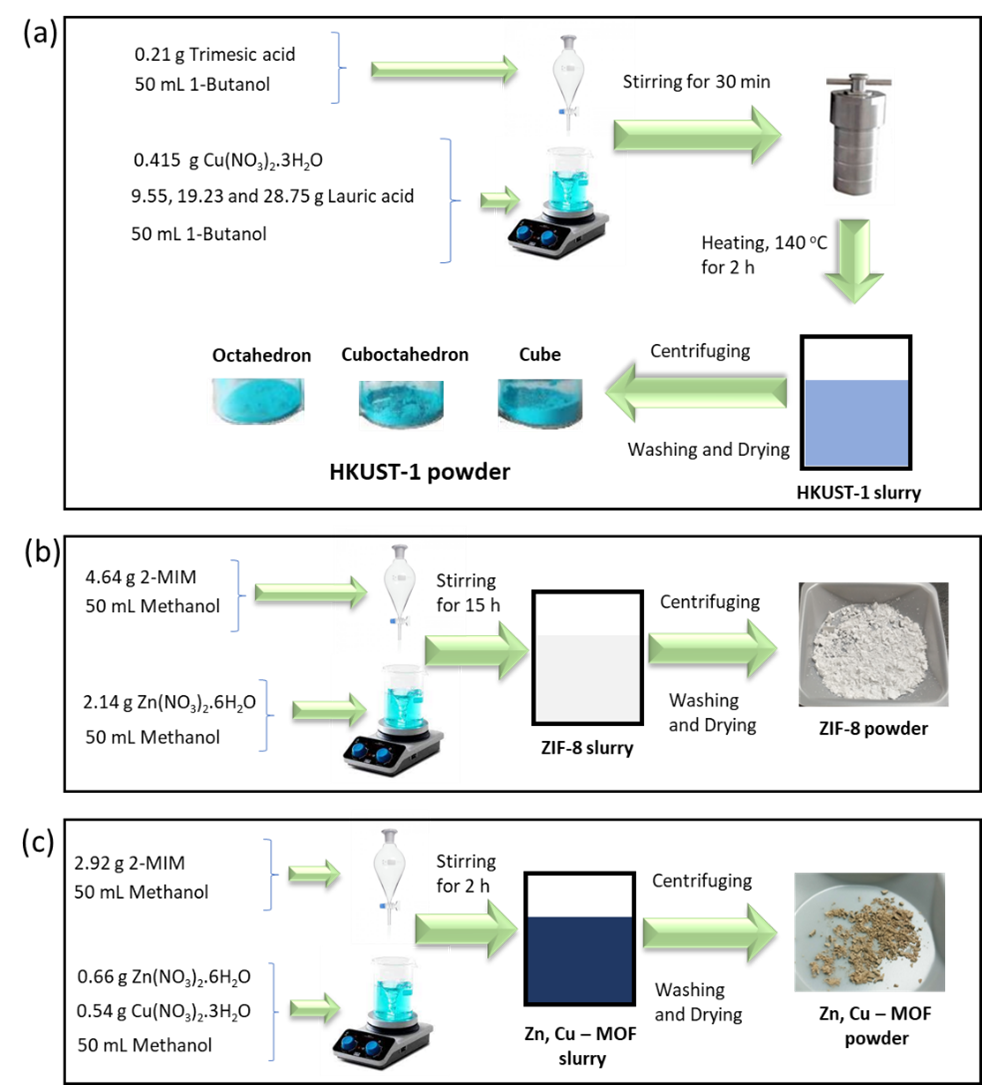


**Figure S20**. Schematic representations of synthesis processes of (a) HKUST-1 with different morphologies, (b) ZIF-8, and (c) Cu/Zn MOF.


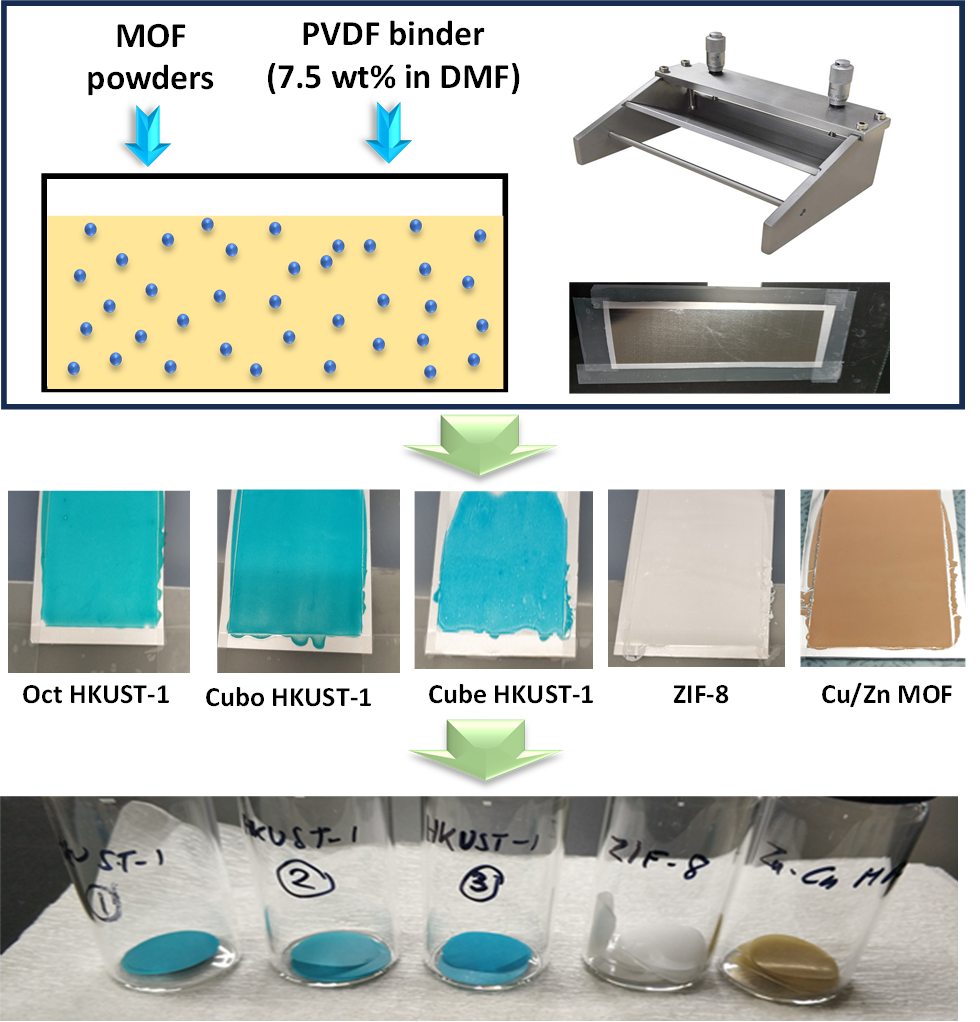


**Figure S21**. Schematic representations of preparation process of MOF films.


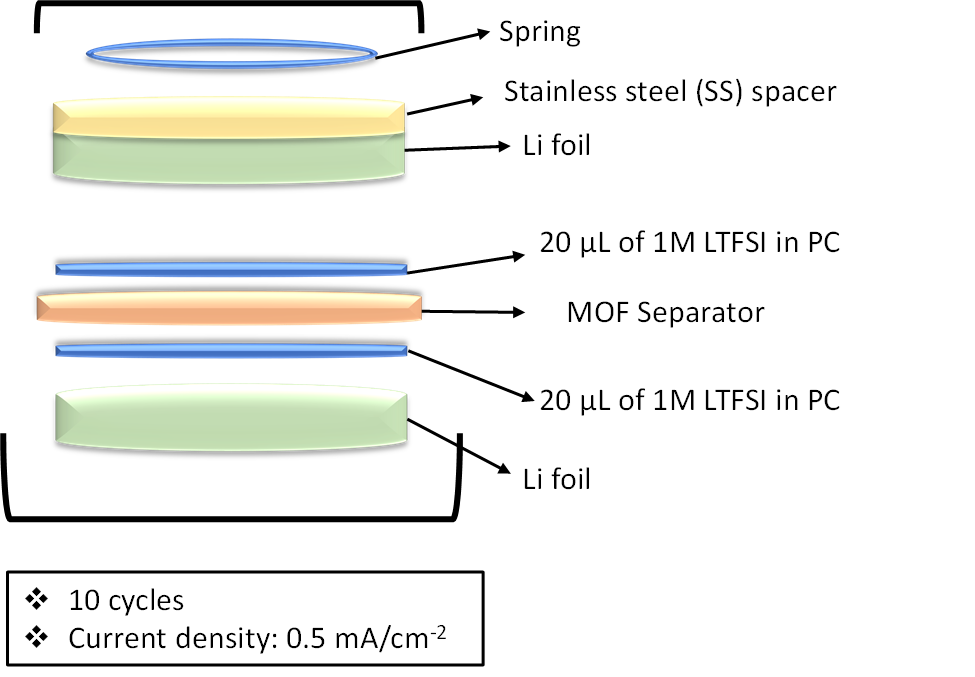


**Figure S22**. Structure configuration of lithium symmetric cells for the electrochemical activation of MOF films.


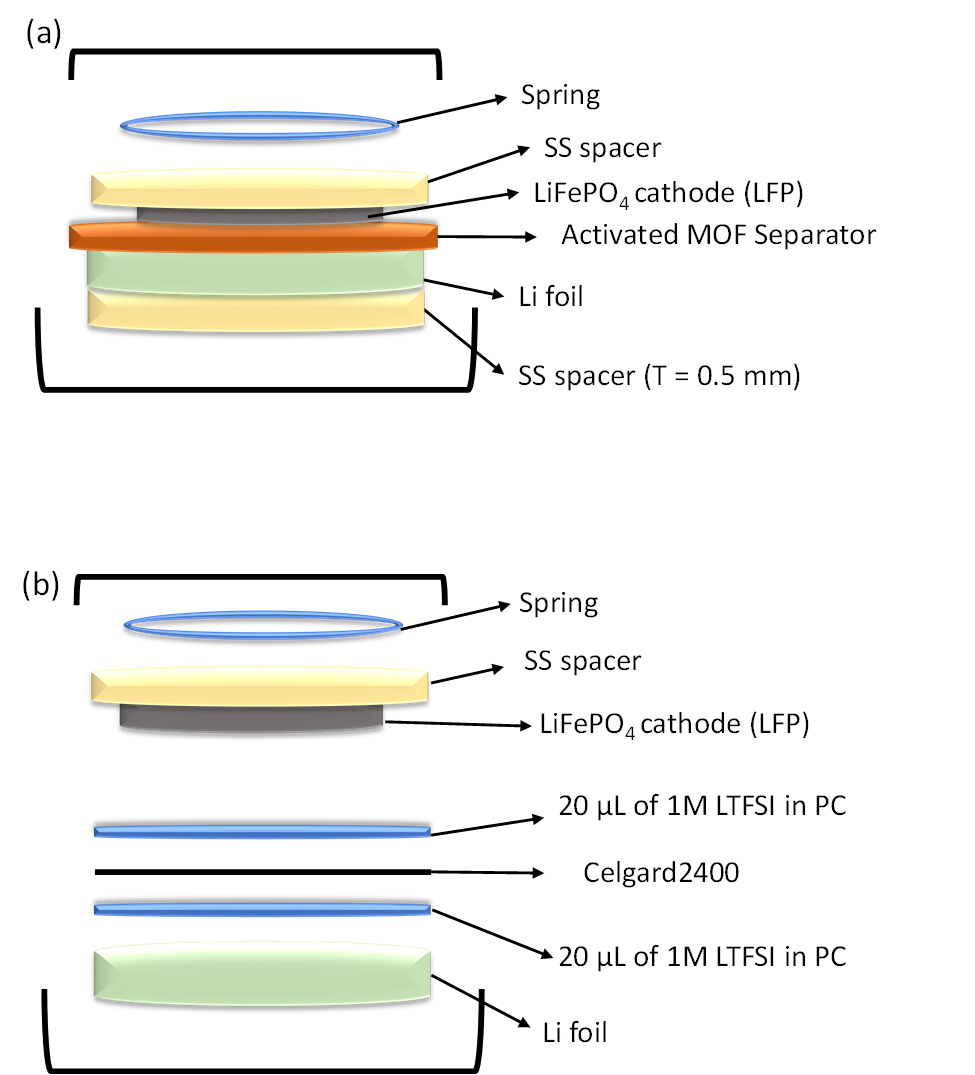


**Figure S23**. Structure configuration of Li//LFP half cells assembled with (a) MOF-based semi-solid electrolytes and (b) typical liquid electrolyte (1M LiTFSI in PC).


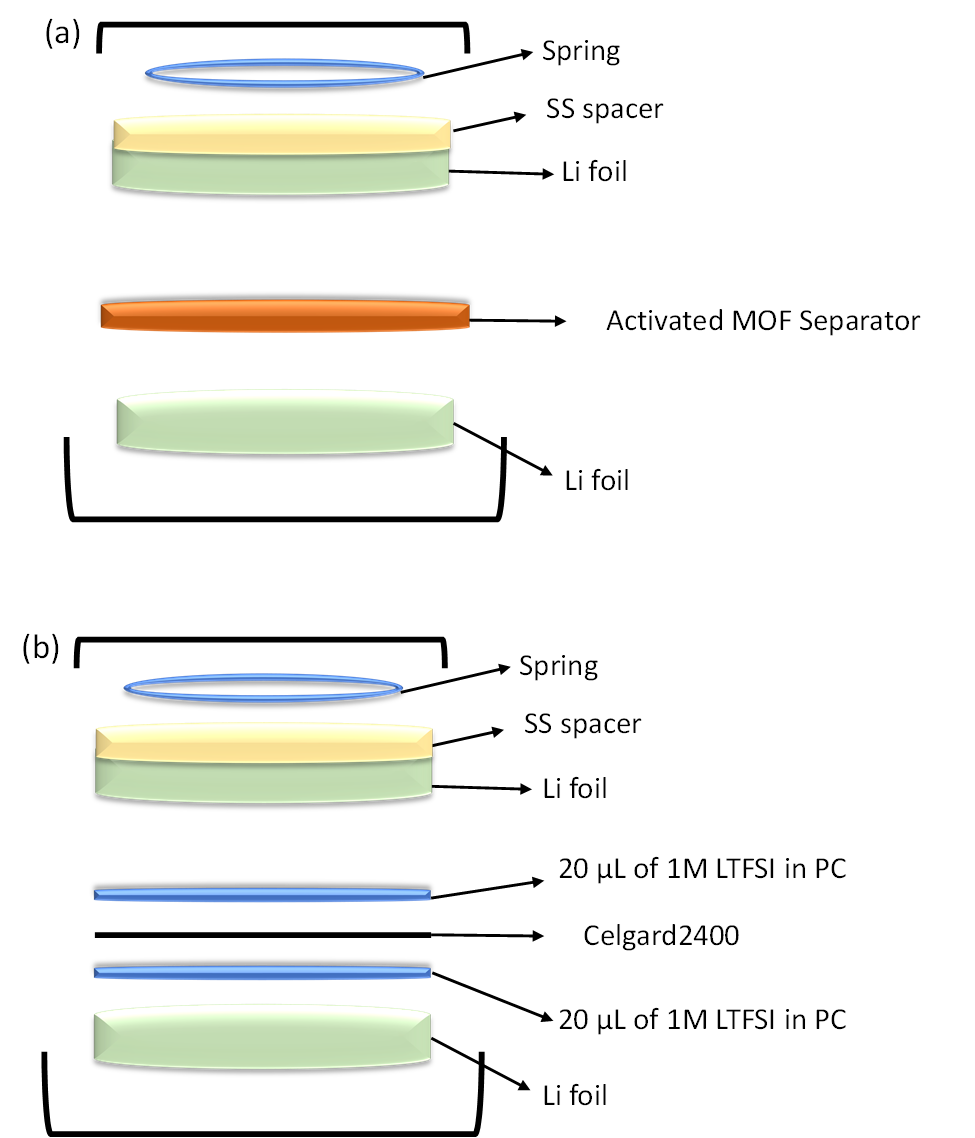


**Figure S24**. Structure configuration of lithium symmetric cells assembled with (a) MOF-based semi-solid electrolytes and (b) typical liquid electrolyte (1M LiTFSI in PC).

**Supplementary References**

[1] B. Mbergen, W. Heitler, E. Teller, P. Roy Soc, E.M. Purcell, H.C. Torrey, R. V Pound, B. Rollin, J. Hatton, A.H. Cooke, R.J. Benzie, Relaxation Effects in Nuclear Magnetic Resonance Absorption, Phys. Rev. 73 (1948) 679. https://doi.org/10.1103/PhysRev.73.679.

[2] P. Dong, X. Zhang, W. Hiscox, J. Liu, J. Zamora, X. Li, M. Su, Q. Zhang, X. Guo, J. McCloy, M.K. Song, Toward High-Performance Metal–Organic-Framework-Based Quasi-Solid-State Electrolytes: Tunable Structures and Electrochemical Properties, Adv. Mater. 35 (2023) 2211841. https://doi.org/10.1002/ADMA.202211841.

[3] K. Jeong, S. Park, G.Y. Jung, S.H. Kim, Y.H. Lee, S.K. Kwak, S.Y. Lee, Solvent-Free, Single Lithium-Ion Conducting Covalent Organic Frameworks, J. Am. Chem. Soc. 141 (2019) 5880–5885. https://doi.org/10.1021/JACS.9B00543/SUPPL_FILE/JA9B00543_SI_001.PDF.

[4] D.A. Vazquez-Molina, G.S. Mohammad-Pour, C. Lee, M.W. Logan, X. Duan, J.K. Harper, F.J. Uribe-Romo, Mechanically Shaped Two-Dimensional Covalent Organic Frameworks Reveal Crystallographic Alignment and Fast Li-Ion Conductivity, J. Am. Chem. Soc. 138 (2016) 9767–9770. https://doi.org/10.1021/JACS.6B05568/SUPPL_FILE/JA6B05568_SI_001.PDF.

[5] J. Evans, C.A. Vincent, P.G. Bruce, Electrochemical measurement of transference numbers in polymer electrolytes, Polymer (Guildf). 28 (1987) 2324–2328. https://doi.org/10.1016/0032-3861(87)90394-6.

[6] A. Ponrouch, E. Marchante, M. Courty, J.M. Tarascon, M.R. Palacín, In search of an optimized electrolyte for Na-ion batteries, Energy Environ. Sci. 5 (2012) 8572–8583. https://doi.org/10.1039/C2EE22258B.

[7] L.A. Martins, L.T. Biosca, J.A. Gómez-Tejedor, J.P. Serra, D.M. Correia, C.M. Costa, S. Lanceros-Méndez, J.L. Gómez Ribelles, I. Tort-Ausina, Influence of the Inclusion of Propylene Carbonate Electrolyte Solvent on the Microstructure and Thermal and Mechanical Stability of Poly(l -lactic acid) and Poly(vinylidene fluoride- co-hexafluoropropylene) Battery Separator Membranes, J. Phys. Chem. C. 127 (2023) 10480–10487. https://doi.org/10.1021/ACS.JPCC.3C02514/ASSET/IMAGES/LARGE/JP3C02514_0005.JPEG.
